# A Guide to Conquer the Biological Network Era Using Graph Theory

**DOI:** 10.3389/fbioe.2020.00034

**Published:** 2020-01-31

**Authors:** Mikaela Koutrouli, Evangelos Karatzas, David Paez-Espino, Georgios A. Pavlopoulos

**Affiliations:** ^1^Institute for Fundamental Biomedical Research, BSRC “Alexander Fleming”, Vari, Greece; ^2^Department of Informatics and Telecommunications, University of Athens, Athens, Greece; ^3^Lawrence Berkeley National Laboratory, Department of Energy, Joint Genome Institute, Walnut Creek, CA, United States

**Keywords:** biological networks, topology, graph theory, visualization, clustering

## Abstract

Networks are one of the most common ways to represent biological systems as complex sets of binary interactions or relations between different bioentities. In this article, we discuss the basic graph theory concepts and the various graph types, as well as the available data structures for storing and reading graphs. In addition, we describe several network properties and we highlight some of the widely used network topological features. We briefly mention the network patterns, motifs and models, and we further comment on the types of biological and biomedical networks along with their corresponding computer- and human-readable file formats. Finally, we discuss a variety of algorithms and metrics for network analyses regarding graph drawing, clustering, visualization, link prediction, perturbation, and network alignment as well as the current state-of-the-art tools. We expect this review to reach a very broad spectrum of readers varying from experts to beginners while encouraging them to enhance the field further.

## Introduction

While most recent review articles focus on biomedical and biological networks and their applications (McGillivray et al., 2018; Sonawane et al., 2019; Yue et al., 2019), in certain case studies, familiarity with the graph theory concepts behind these networks is often missing. The aim of this review is to tackle questions raised by today's increasing demands and aid researchers in understanding the graph theory behind the biomedical networks as well as concepts such as visualization, annotation, management, clustering, integration, etc. To do this, we start with an introduction about graphs (in discrete mathematics) and their different types and we further describe the various data structures and file formats for storage and representation. In addition, we discuss several topological features and network properties, as well as concepts such as graph clustering, clustering comparison, network alignment, motif detection, and edge prediction. We further comment on the various layout and graph drawing techniques as well as on methods regarding network alignment and link predictions and we highlight the state-of-the-art tools for analyzing such networks. Finally, we try to bring graph theory into a biomedical context by providing a thorough description about the different types of biomedical networks and the sources used for their construction. We hope this review becomes a useful handbook for readers regardless of their scientific background and help non-experts in handling and interpreting networks more easily.

In general, networks or graphs (mathematical way of representing a network) are used to capture relationships between entities or objects. In a typical representation, a graph is composed of a set of vertices/nodes/points, connected with edges/lines/links/arrows/arcs. Examples of networks which we interact with in everyday life include the electricity grid, road maps, the world wide web, the internet, airline connections, citation and language networks, telecommunication channels, social networks, economic networks, and many others. Graph theory has been the established mathematical field for the study and the analysis of such networks and is applicable to a wide variety of disciplines, ranging from mathematics, physics, computer science, engineering, and sociology to biology and medicine (Junker and Schreiber, 2008; Pavlopoulos et al., 2011a). In the biomedical field for example, many biological networks consist of molecules such as DNA, RNA, proteins and metabolites, and graphs can be used to capture the interactions between these molecules. Therefore, it is essential to know the various network types which can be used, in order to be able to communicate and visualize such interactions.

**Figure 1:**
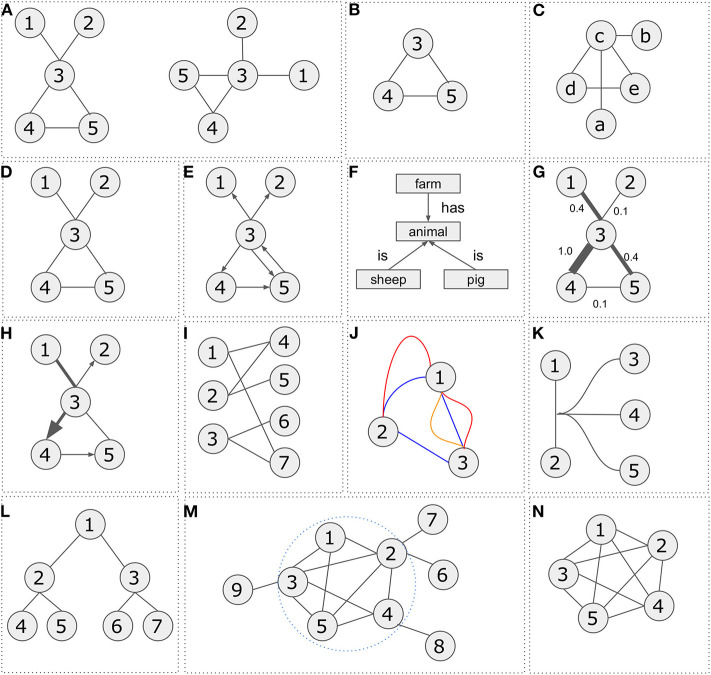
Network representations and types. **(A)** Two graphical representations of a graph *G* = (*V, E*) with vertex set *V* = {1, 2, 3, 4, 5} and edge set *E* = {{1, 3}, {2, 3}, {3, 4}, {3, 5}, {4, 5}}. **(B)** Representation of subgraph *G*′ = (*V*′, *E*′) with vertex set *V* = {3, 4, 5} and edge set *E* = {{3, 4}, {3, 5}, {4, 5}}. **(C)** Graph *G*″ = (*V*″, *E*″) is isomorphic to graph *G* = (*V, E*) with vertex set *V* = {*a, b, c, d, e*} and edge set *E* = {{*a, c*}, {*b, c*}, {*c, d*}, {*c, e*}, {*d, e*}}. **(D)** Undirected graph *G* = (*V, E*) with vertex set *V* = {1, 2, 3, 4, 5} and edge set *E* = {{1, 3}, {2, 3}, {3, 4}, {3, 5}, {4, 5}}. **(E)** Directed graph *G* = (*V, E*) with vertex set *V* = {1, 2, 3, 4, 5} and edge set *E* = {{3, 1}, {3, 2}, {3, 4}, {4, 5}, {3, 5}, {5, 3}}. **(F)** Semantic graph. **(G)** Weighted graph *G* = (*V, E*) with vertex set *V* = {1, 2, 3, 4, 5} and edge set *E* = {{3, 1, 0.4}, {3, 2, 0.1}, {3, 4, 1.0}, {4, 5, 0.1}, {3, 5, 0.4}}. **(H)** Mixed graph *G* = (*V, E*) with vertex set *V* = {1, 2, 3, 4, 5} and edge set *E* = {{1, 3}, {3, 2}, {3, 4}, {5, 3}, {4, 5}}. **(I)** Bipartite graph with vertex set *V*′ = {1, 2, 3}, *V*″ = {4, 5, 6, 7} and edge set *E* = {{1, 4}, {1, 7}, {2, 4}, {2, 5}, {3, 6}, {3, 7}}. **(J)** Multi-edge graph *G* = (*V, E*) with vertex set *V* = {1, 2, 3} and three different types of edge sets *E*′ = {{1, 2}, {2, 3}, {3, 1}}, *E*″ = {{1, 2}, {1, 3}}, *E*^‴^ = {{1, 3}}. **(K)** Hypergraph *G* = (*V, E*) with vertex set *V* = {1, 2, 3, 4, 5} and an edge connecting multiple nodes *E* = {{1, 2, 3, 4, 5}}. **(L)** A tree graph *G* = (*V, E*) with vertex set *V* = {1, 2, 3, 4, 5, 6, 7} and edge set *E* = {{1, 2}, {2, 4}, {2, 5}, {1, 3}, {3, 6}, {3, 7}}. **(M)** A graph *G* = (*V, E*) with vertex set *V* = {1, 2, 3, 4, 5, 6, 7, 8, 9} and edge set *E* = {{1, 2}, {1, 3}, {1, 5}, {2, 3}, {2, 4}, {2, 5}, {2, 6}, {2, 7}, {3, 4}, {3, 5}, {3, 9}, {4, 5}, {4, 8}}. A cluster consisting of nodes *V* = {1, 2, 3, 4, 5} and edges *E* = {{1, 2}, {1, 3}, {1, 5}, {2, 3}, {2, 4}, {2, 5}, {3, 4}, {3, 5}, {4, 5}}. **(N)** A five-node clique on the right. Any node is connected with any other node.

Starting with the basic notions, in mathematics, a *set* *A* = {*a*_1_, *a*_2_, *a*_3_, …*a*_*n*_} is a collection of objects *a*_1_, *a*_2_, *a*_3_, …*a*_*n*_, whereas a *graph* *G* = (*V, E*) is composed of a set of *vertices* *V* and a set of *edges* *E*. A *subgraph* *G*′ = (*V*′, *E*′) of the graph *G* = (*V, E*) is a graph where *V*′ is a subset of *V* and *E*′ a subset of *E*. While one graph can have multiple representations, two different graphs may be *isomorphic* if they contain the same number of vertices connected in the same way. Examples are shown in Figures 1A–C.

There are various graph categories. The most known are *undirected, directed, weighted, bipartite, multi-edge, hypergraphs*, and *trees*.

A graph is ***undirected*** if there is a single connection defined as *E* = {(*i, j*)|, *i, j* ∈ *V*} between vertices *i* and *j*. In such case, vertices *i* and *j* are called direct neighbors (e.g., gene co-expression network).

A graph is called ***directed*** if an edge between vertices *i* and *j* is represented by an arrow, thus indicating a direction from vertice *i* to vertice *j* or vice versa. A directed graph is defined as an ordered triple *G* = (*V, E, f*) where *f* is a function that maps each element in set *E* to an ordered pair of vertices in *V* (e.g., pathway).

Notably, in biology there are a number of directed relationships which can be graphically shown as different arrow types toward a ***semantic*** approach (e.g., food web). For example, “inhibits,” “enhances,” “regulates” etc. Standards for arrow usage are described in the Systems Biology Graphical Notation (SBGN) visual language (Le Novère et al., 2009).

A ***weighted graph*** is defined as a graph where *E* is a set of edges between the vertices *i* and *j* (*E* = {(*i, j*) | *i, j* ∈ *V*}) associated with a weight function *w*:*E* → *R*, where *R* denotes the set of all real numbers. Most of the times, the weight *w*_*ij*_ of the edge between nodes *i* and *j* represents the relevance of the connection (e.g., sequence similarity network).

A ***bipartite graph*** is an undirected graph *G* = (*V, E*) in which vertices in *V* can be partitioned into two sets *V*′ and *V*″ such that (*i, j*) ∈ *E* implies either (*i* ∈ *V*′ and *j* ∈ *V*″) or (*j* ∈ *V*′ and *i* ∈ *V*″) (e.g., gene-disease networks). In other words, any vertex from set *V*′ can be connected to any other vertex from set *V*″ but no edges between vertices within the same set (*V*′or *V*″) are allowed.

A graph is called ***multi-edge*** if it contains multiple edges or otherwise parallel edges that are incident to the same two vertices (e.g., knowledge/integration networks). A simple graph for example, has no multiple edges.

A ***hypergraph*** consists of a set of vertices V and a set of hyperedges E where an edge can join any number of vertices (e.g., biochemical networks).

A ***tree*** is an undirected graph in which any two vertices are connected by exactly one path, or equivalently a connected acyclic undirected graph (e.g., ontologies, phylogenies). Examples of the various graph types are shown in Figures 1D–L.

A graph is ***connected*** if there is a path from any point to any other point in the graph. In a ***complete graph***, every pair of distinct vertices is connected by a unique edge.

A ***cluster*** (Figure 1M) is a graph formed from the disjoint union of complete graphs and a ***clique*** (Figure 1N) in an undirected graph is a subset of vertices such that every pair of vertices in the clique is connected.

## Data Structures and Representations

**Figure 2:**
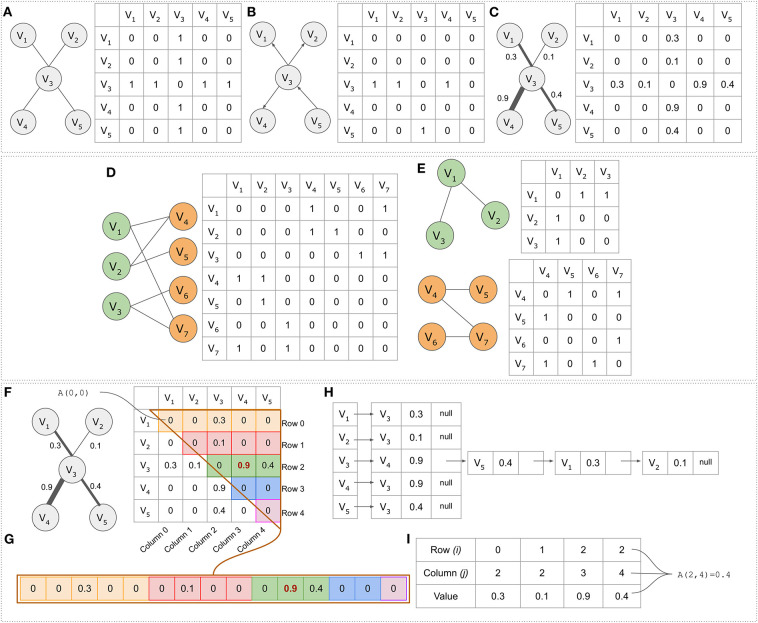
Adjacency matrices and alternative data structures. **(A)** Simple undirected graph consisting of five nodes (*N* = *V* = 5) and four edges (*E* = 4). **(B)** A directed graph represented by a non-symmetric adjacency matrix. **(C)** A simple weighted graph. **(D)** The bipartite graph and its adjacency matrix. **(E)** The graph's projections. In the projected network colored as green, node *V*_1_ for example is connected to node *V*_2_ through node node *V*_4_. **(F)** The upper triangular part of the adjacency matrix. **(G)** The upper triangular part of the adjacency matrix in a linear form. Element *A[2,3]* = *0.9* in the adjacency matrix is element *B[10]* = *0.9* in the linear form. **(H)** The graph presented as an adjacency list. Each vertex is accompanied by a list containing all other vertices adjacent to it. **(I)** A data structure for efficiently storing sparse matrices with many zeros. The first two rows indicate the coordinates in an adjacency matrix, whereas the third column contains the connection weight.

A network can be stored as **(i) adjacency matrix, (ii) adjacency list, or (iii) sparse matrix**. In graph theory, an adjacency matrix *A* is a square matrix of size *N* × *N* (where *N* is the number of vertices) used to represent a graph. In the case of a simple graph, the adjacency matrix is a (Sabidussi, 1966; Yue et al., 2019)-matrix with zeros on its diagonal (*A[i,j]* = 1 for connection presence, *A[i,j]* = 0 for connection absence) or a (0,*w*_*ij*_)-matrix for a weighted graph where *w*_*ij*_ is the edge weight between two nodes (*A[i,j]* = *w*_*ij*_). In both undirected simple and weighted graphs, the adjacency matrix is symmetric (equal to its transpose-rows and columns are the same). In the case of directed graphs, the matrix is not symmetric, thus differentiating its upper triangular part from its lower triangular part (*ij* is not the same as *ji*). An overview of adjacency matrices and their representations are shown in Figures 2A–C.

Bipartite graphs, as opposed to generic networks, have their own characteristics (Pavlopoulos et al., 2018). One major property is that any bipartite graph can be presented as two biadjacency matrices (or otherwise projections). While in an original bipartite graph, vertices which belong to a set are not connected to each other, in its biadjacency form they are connected through nodes that belong to the other set (indirect connections). This concept is described in Figures 2D,E, whereas an extensive review about their biomedical application can be found elsewhere (Pavlopoulos et al., 2018).

Adjacency matrices are memory inefficient for storing larger sparse networks as they require *O*(*V*^2^) memory. Notably, the *O* notation in graph theory is a theoretical measure to classify algorithms according to how their running time or space requirements grow as the input size grows (Knuth, 1997). Let's assume that in a gene co-expression network, one wants to store an all-vs.-all matrix with all pairwise human gene similarities (*V* = ~20,000 genes). This would require 400,000,000 bytes to be stored in memory (381 MB RAM) or $\frac{4\times {20,000}^{2}}{{1,024}^{3}}=1.49\;GB$ for float/integer numbers (for 4 byte integers and floats). To partially overcome this barrier, a simple approach would be to take advantage of the adjacency matrix symmetry by only storing the upper triangular part in an array *B* in a linear form (Figures 2F,G). The mapping between element coordinates in the two forms is given by the formula $A\left[i,j\right]=B\left[Ni+\frac{i\left(1-i\right)}{2}+\left(j-i\right)\right]$ where *N* is the number of vertices (Figure 2G). The linear representation *B* requires $\frac{V\left(V-1\right)}{2}$ memory which is half the size compared to the memory needed for a complete adjacency matrix *A*.

For sparse networks, adjacency lists are proposed as an alternative data structure. An adjacency list is an array *A* of separate lists. Each element of the array *A*_*i*_ is a list, which contains all the vertices that are adjacent to vertex *i*. If the graph *G* is weighted, then each item in the adjacency list is either a two-item array or an object, giving the vertex number, and the edge weight (Figure 2H). Adjacency lists require much less space *O*(*V*+*E*) compared to the space required by the adjacency matrix *O*(*V*^2^). Moreover, finding all vertices adjacent to a given vertex in an adjacency matrix representation, requires *O*(*V*) time, whereas in an adjacency list such operation is as fast as reading the corresponding list (smaller length).

An alternative to the adjacency list, is the use of a sparse matrix data structure. In such case only the non-zero elements are kept along with their coordinates and everything else is discarded as non-informative. An example of such a data structure is shown in Figure 2I where the first row keeps the *i* coordinate for each element in *A*[*i, j*], the second row the *j* coordinate in *A*[*i, j*] and the third row the weight *w*_*ij*_. In the case of unweighted simple graphs (referring to the default value which equals to 0, indicating that no link exists), the third row can be completely skipped, remembering that *w*_*ij*_ is always one.

## General Network Properties

**Figure 3:**
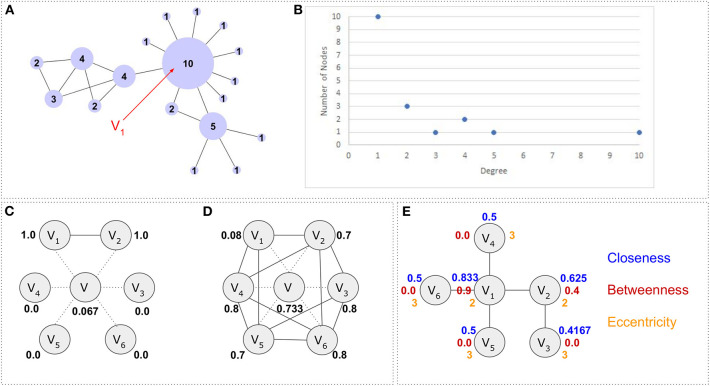
Network properties and topological features. **(A)** A network *G* = (*V, E*) consisting of V = 18 nodes and E = 21 edges. Each node's size has been adjusted according to its degree. Vertex *V*_1_ for example has 10 neighbors, thus degree *d*(*V*_1_) = 10. The average degree for the whole network is $\frac{42}{18}=2.333$. Network has been visualized with Cytoscape. **(B)** A scatterplot histogram showing the degree distribution. The Y axis holds the values about how many nodes have certain degree (values in X axis). **(C)** Clustering coefficient. Node *V* has 6 neighbors {*V*_1_ *V*_2_, *V*_3_, *V*_4_, *V*_5_, *V*_6_}. The maximum number of edges between these neighbors are $\frac{6\left(6-1\right)}{2}=$15 but only two neighbors (V_1_ and V_2_) are connected to each other thus making the clustering coefficient for node *V* equal to $\frac{1}{15}=0.066$. **(D)** Similarly, the neighbors of node *V* are connected with 11 edges between each other (E = {{V_1_,V_2_}, {V_1_,V_5_}, {V_1_,V_4_}, {V_2_,V_3_}, {V_2_,V_6_}, {V_2_,V_4_}, {V_3_,V_6_}, {V_3_,V_5_}, {V_4_,V_6_}, {V_4_,V_5_}, {V_5_,V_6_}}), the clustering coefficient for this node will be $C_{V}=\frac{11}{15}=\;$0.733. Notably dotted lines represent the direct connections of node *V*, whereas the solid lines represent the connections between the first neighbors of node *V*. **(E)** The closeness centrality in blue, the betweenness centrality in red and the eccentricity centrality in orange. The graph consists of 6 nodes and 5 edges. *Closeness centrality* calculation example: Node *V*_1_accesses nodes *V*_2_, *V*_4_, *V*_5_, *V*_6_ with step 1 and node *V*_3_ with step 2. Therefore, its closeness centrality is calculated as $\frac{5}{4\times 1+2\times 1}=\frac{5}{6}=0.833$. *Betweenness centrality* calculation example: Since all nodes are accessible through any other node, there are *N*(*N* − 1) = 6 × 5 = 30 shortest paths but only 12 of them pass through node *V*_2_. These are {*V*_3_, *V*_2_}, {*V*_3_, *V*_2_, *V*_1_}, {*V*_3_, *V*_2_, *V*_1_, *V*_4_}, {*V*_3_, *V*_2_, *V*_1_, *V*_5_}, {*V*_3_, *V*_2_, *V*_1_, *V*_6_}, {*V*_2_, *V*_1_}, {*V*_2_, *V*_1_, *V*_4_}, {*V*_2_, *V*_1_, *V*_6_}, {*V*_2_, *V*_1_, *V*_5_}, {_*V*_4_, *V*1_, *V*_2_, *V*_3_}, {_*V*_5_, *V*1_, *V*_2_, *V*_3_} and {_*V*_6_, *V*1_, *V*_2_, *V*_3_}. Therefore the $C_{bet\left(V_{2}\right)}=\frac{12}{30}=0.4$. *Eccentricity* calculation example: Node *V*_1_ accesses nodes *V*_2_, *V*_4_, *V*_5_, *V*_6_ with one step and node *V*_3_ with two steps. Therefore, its eccentricity will be *max* (2, 1) = 2.

As ***degree*** *deg*_*i*_, we define the total number of edges adjacent to a vertex. In the case of a directed graph we distinguish between the “*indegree”* ($deg_{i}^{in}$) and “*outdegree”* $\left(deg_{i}^{out}\right)$. The indegree refers to the number of arcs, incident from the vertex, whereas the outdegree to the number of arcs incident to the vertex. In a social network for example, the indegree would represent the followers, whereas the outdegree the people one follows. The total degree in a directed graph is the sum of the indegree and outdegree $deg_{i}=deg_{i}^{in}+deg_{i}^{out}$ showing all connections (both followers and followed people). The average degree of the network is $deg_{avg}=\frac{\Sigma deg_{i}}{V}$ (Figure 3A). Looking at all nodes in a network, in order to study the *degree distribution* *p*(*k*), we consider the probability that a randomly selected vertex has degree equal to *k*. The same information can also be found as *cumulative degree distribution* *p*_*c*_(*k*) which shows the a-posterior probability of a randomly selected vertex to have degree larger than *k*. Notably, the degree distribution is one of the most important topological features and is characteristic to different network types. In the simplest case, *p*(*k*) can be estimated by a histogram of degrees. An example is shown in Figure 3B. Networks, whose degree distribution follow a power law, are called ***scale-free*** networks.

***Density*** is the ratio between the number of edges in a graph and the number of possible edges in the same graph. In a fully connected graph (e.g., protein complex), the number of possible edges (pairwise connections) are $E_{max}=\frac{V\left(V-1\right)}{2}$. Therefore, the density can be calculated as $density=\frac{E}{E_{max}}=\frac{2E}{V\left(V-1\right)}$. If a graph has *E* ≃ *V*^*k*^, 2 > *k* > 1, then this graph is considered as *dense*, whereas when a graph has *E* ≃ *V* or *E* ≃ *V*^*k*^, *k* ≤ 1, it is considered as *sparse*.

The ***Clustering coefficient*** is a measure which shows whether a network or a node has the tendency to form clusters or tightly connected communities (e.g., protein clusters in a protein-protein interaction network). The clustering coefficient of a node is defined as the number of edges between its neighbors divided by the number of possible connections between these neighbors. The clustering coefficient of a node *i* is defined as $C_{i}=\frac{2e}{k\left(k-1\right)}$ where *k* is the number of neighbors (degree) and *e* the number of edges between these *k* neighbors. The average clustering of a network is given by $C_{avg}=\frac{\Sigma C_{i}}{V}$. The clustering coefficient takes values 0 ≤ *C*_*i*_ ≤ 1, thus the closer to 1, the higher the tendency for clusters to be formed. An example is shown in Figures 3C,D.

The ***matching index*** *M*_*ij*_ can be used to identify two nodes in a network which might be functionally similar without necessarily being connected to each other. The matching index is a measure to quantify such similarity between any two nodes within a network and, according to the above, two nodes can be found to be functionally similar if they share common neighbors. The matching index between vertices *i* and *j* is calculated as $M_{ij}=\frac{\Sigma \;distinct\;common\;neighbors}{\Sigma \;total\;number\;of\;neighbors}$ and can be extended beyond the direct neighbors of a vertex. In addition, it can be applied to multi-edge networks.

The ***distance*** *dist*_*ij*_ between two nodes (e.g., metabolites in a metabolic network) is defined as the length of the *shortest path* between them. As shortest path we define the minimal number of edges that need to be traversed to reach node *j* from node *i*. In the case where two shortest paths of identical length exist, any of them could be used. Whenever there is no connection between two nodes *i* and *j*, then their distance is defined as infinite *dist*_*ij*_ = ∞. In addition, the *diameter*, *diam*_*m*_ = *max*(*dist*_*ij*_), is the maximal distance between any pair of vertices. The average path length is defined as the average distance between all node pairs and is defined as $dist_{avg}=\frac{1}{N\left(N-1\right)}\overset{N}{\underset{i\;=\;1}{\sum }}\overset{N}{\underset{j\;=\;1}{\sum }}dist_{ij}.$

## Network Centralities

Very often, in network analysis, we ask questions such as: which is the most important node, which node behaves as a hub, which node is the bridge between two different communities, which node is important for the network's robustness (tolerance to failures and perturbations), etc. In order to address these questions, various network centralities can be used. The ***degree centrality*** (Bonacich, 1987) is a measure to highlight highly connected nodes (e.g., central transcription factors). A network with a star-like topology for example, contains hubs which are central nodes with many neighbors around them. The degree centrality of a node *i* is calculated as *C*_*i*_ = *deg*(*i*) where *deg*(*i*) is the node's degree. Similarly, the ***closeness centrality*** (Sabidussi, 1966) is a measure to detect important nodes which can communicate quickly with other nodes in a network. For a graph *G* = (*V, E*) it is defined as $C_{clo}=\frac{1}{\sum dist_{ij}}$ or as $C_{clo}=\frac{N-1}{\sum dist_{ij}}$ in its normalized form. In biochemical networks, it is often used to find top metabolites [e.g., metabolites in *E. coli* as part of the glycolysis and citrate acid cycle pathways (Ma and Zeng, 2003; Koschützki and Schreiber, 2008)]. The ***betweenness centrality*** (Freeman, 1977) shows the nodes which form such bridges so that two communities can communicate with each other. It is calculated as $C_{bet\;\left(i\right)}=\frac{\sigma_{xy}\left(i\right)}{\sigma_{xy}}$ where σ_*xy*_ is the total number of shortest paths from node *x* to node *y* and σ_*xy*_(*i*) is the number of those paths that pass through node *i*. It has been shown that proteins with high betweenness centrality in a protein-protein interaction (PPI) network play an important role to the modularization of the network (Koschützki and Schreiber, 2008). The ***eccentricity centrality*** (Hage and Harary, 1995) shows how easily accessible a vertex is from any other vertex in the network. The *eccentricity* is the maximum graph distance between vertex *i* and any other vertex *j* in graph *G*. For a disconnected graph, all vertices are defined to have infinite eccentricity. The *eccentricity centrality* is calculated as $C_{ecc}=\frac{1}{max\left(dist_{ij}\right)}$. Eccentricity centrality has been used to detect essential proteins in a PPI (Jalili et al., 2016). Notably, the maximum eccentricity is called the graph ***diameter***, whereas the minimum graph eccentricity is called the graph ***radius***. Finally, there are many other specialized centralities that serve different purposes. The ***eigenvector centrality*** for example, detects vertices that are connected to important vertices, whereas the ***subgraph centrality*** accounts for the participation of a node in all subgraphs of the network. Examples are shown in Figure 3E.

## Motifs

Network motifs are repeated graphlets (small subgraphs of a larger network that appear at any frequency) in a specific network capturing particular patterns of interactions between vertices. They are often associated with particular functions (Stone et al., 2019) and are used for many applications in biological networks (Kim et al., 2011). Motifs are structures which occur at higher frequencies compared to random networks and are found in both directed and undirected networks. Motif analysis is often applied on biological networks such as biochemical, ecological, neurobiology, or gene expression networks to unravel building blocks associated with certain biological processes.

**Figure 4:**
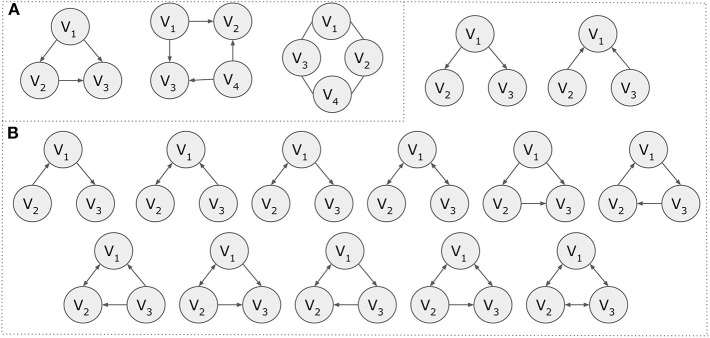
Motifs. **(A)** Motif examples of three and four nodes. **(B)** The 13 possible directed motifs using three nodes.

For example, motifs can be found in ecological food webs as well as genetic networks or the World Wide Web (Milo, 2002; Shen-Orr et al., 2002). Feed-forward-loop (FFL) and bifan motifs (Figure 4) are typical patterns found in various types of biological networks (Mangan and Alon, 2003; Mangan et al., 2003). Notably, motifs have been used to distinguish different protein-protein interaction networks (Przulj et al., 2004) and in contrast to the transcriptional regulatory networks, it has been shown that they are evolutionary conserved in PPI networks (Conant and Wagner, 2003).

To measure the statistical significance of a network motif, a *Z*-score or a *P*-value can be used. The *Z*-score is calculated as the difference of the frequency *f*(*m*) of a motif *m* in a network and its mean frequency *f*_*r*_(*m*) in a large number of randomized networks σ_*r*_(*m*). The formula is $Z\left(m\right)=\frac{f\left(m\right)-f_{r}\left(m\right)}{\sigma_{r}\left(m\right)}$. Similarly, the *P*-value shows the probability *P*(*m*) of a motif *m* to appear in a randomized network equally or more times than in the network of interest. Motifs are considered to be statistically significant if they have *Z*(*m*) > 2.0. Motif detection can become computationally expensive and tools like Pajek (Mrvar and Batagelj, 2016), Mfinder (Kashtan et al., 2004), MAVisto (Schreiber and Schwöbbermeyer, 2005), NetMatch (Ferro et al., 2007), SANA (Mamano and Hayes, 2017), and FANMOD (Wernicke and Rasche, 2006) are offered for this purpose (Kavurucu, 2015).

## Models

**Figure 5:**
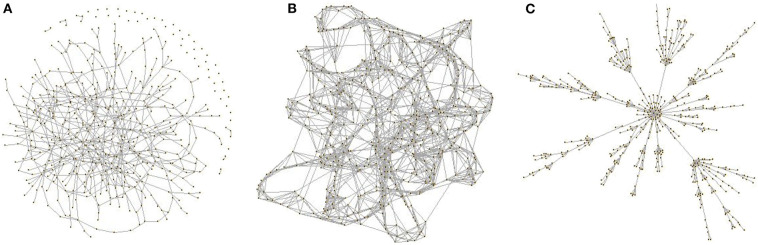
Network models. **(A)** An Erdos–Rényi random network. **(B)** A Watts-Strogatz network. **(C)** Barabási–Albert (BA) scale-free network. Graphs were visualized using R. Code example: *g1* = *sample_smallworld (1, size* = *500, nei* = *4, p* = *0.03). Plot (g1, layout* = *layout.fruchterman.reingold, vertex.label* = *NA, edge.arrow.size* = *0.02, vertex.size* = *0.5, xlab* = “*Random Network: G(N,p) model”)*.

In order to better understand a network's topology and come to the conclusion of whether observed features are network-specific or not, several models such as the *Erdos–Rényi* (Bollobás, 2001), *Watts-Strogatz* (Watts and Strogatz, 1998), and *Barabási–Albert* (Barabasi and Albert, 1999) have been introduced (Figure 5).

The ***Erdos–R*é*nyi*** model: It is one of the most popular models in graph theory and was mainly introduced to describe the properties of a random graph. According to this model, *V* number of vertices are randomly connected with probability $p=\frac{2E}{V\left(V-1\right)}$. In general, in such a graph, each pair of vertices can be connected with approximately an equal probability *p* ≤ 1, whereas the degree distribution is given by a binomial distribution. The probability of a vertex to have degree *deg* is $p\left(deg\right)≃e^{-deg_{avg}}\frac{{deg_{avg}}^{deg}}{deg!}$. Notably, for a network where *V* → ∞ the distribution becomes approximately Poissonian. A typical characteristic of a random network is its homogeneity as most vertices have a similar number of connections. For small *p*, the network seems as disconnected, whereas for $p\approx \frac{1}{V}$, the network has a bigger component containing most of the network's connections. When $p\geq \frac{log\left(V\right)}{V}$, then almost all vertices are connected homogeneously and at random. The clustering coefficient of this network is $C=p=\frac{deg_{avg}}{V}$ and shows that the probability of two nodes with a common neighbor to be connected is the same as the probability of two randomly paired vertices. In the case of biological networks, straightforward comparisons show if they have a certain topology or differ from any other random network. Thus, Erdos–Rényi is not a good model for biological networks with respect to degree distribution.

The ***Watts-Strogatz*** model: This model was introduced to describe random networks that follow a small world topology meaning that most nodes can be reached by any other node in a small number of steps. While random networks can often capture this property too, they fail to account for highly connected regions like in most empirical networks (e.g., social networks). Therefore, Watts and Strogatz proposed a model for networks described by local structures (high clustering coefficient) as well as small average path lengths. Metabolic networks [e.g., fat-metabolism communication in Yeast (Al-Anzi et al., 2015)], in which metabolites are linked to each other with small steps, is a typical example (Jeong et al., 2000). In a Watts-Strogatz network, if all vertices are placed on a circular ring, each vertex would be connected to its $\frac{V}{2}$ neighbors. In the real world, this indicates the form of small communities where people know other people from their close environment as well as friends of friends from nearby areas. Coexistence of high local clustering and short average path length are two main characteristics of this type of networks.

The ***Barab*á*si–Albert*** model: This model describes random ***scale-free*** networks. These are networks whose degree distribution follows a power law taking into account their inhomogeneous degree distribution or otherwise networks with nodes which do not have a typical number of neighbors. According to this model, networks can evolve overtime and new edges do not appear randomly, whereas new nodes follow the existing degree distribution. At time point *t* = 0 for example, let's assume a network consisting of *V*_0_ vertices and zero edges. A new vertex will connect with *e* ≤ *V*_0_ edges to the existing vertices, whereas after *t* time points, the network is expected to consist of *V* = *V*_0_ + *et* edges. Notably, for *t* ≫ 1, the Barabasi-Albert model will exhibit a scale-free distribution *p*(*k*) ~ *k*^−γ^, γ = 3. Like in a social network, individuals who already have many friends are likely to acquire more friends overtime compared to individuals with a limited number of friends. When comparing the Erdos–Rényi and Watts-Strogatz networks of the same size and density, the Barabasi-Albert networks were found to have shorter average path lengths. Characteristic examples of BA networks are the Protein-Protein interaction networks (Barabási and Oltvai, 2004; Yook et al., 2004).

Like in many real-life examples, most biological networks are robust and tolerant against random removal of nodes as biological functions must remain maintained. However, compared to random networks with homogeneous degree distribution, scale-free networks are very vulnerable to targeted attacks but very robust against random removal of vertices. In general, nodes with low degree appear more frequently compared to nodes with high degree and play a minor role in the overall network topology, whereas aimed removal of nodes with higher degree distribution can affect a network's topology significantly.

## Biological and Biomedical Networks

**Figure 6:**
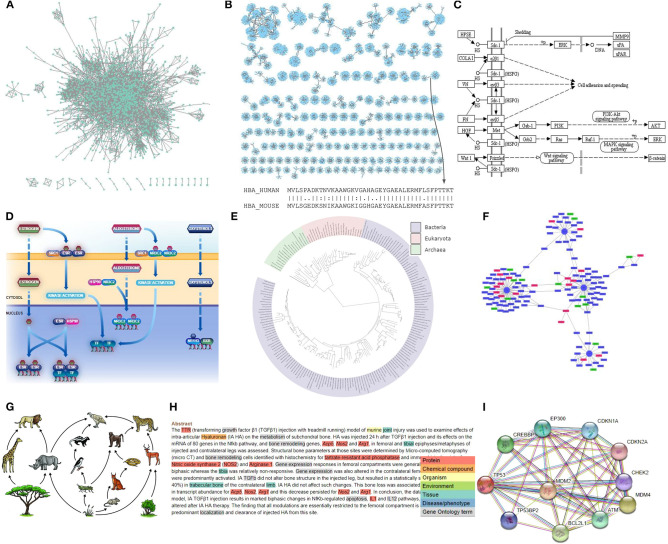
Examples of biological networks. **(A)** A protein-protein interaction (PPI) network shown in Cytoscape. **(B)** A sequence similarity network visualized with Cytoscape. Each edge corresponds to an alignment score. **(C)** A KEGG metabolic pathway. **(D)** A Reactome signal transduction network. **(E)** The tree of life visualized by iTOL. **(F)** A gene expression network with up- (red) and down-regulated genes (green). **(G)** A Savanna food web (credit: Siyavula Education). **(H)** A tagged PubMed abstract showing abstract-based co-occurrences. **(I)** A STRING multi-edge PPI knowledge network.

In biomedical research, graphs can capture the associations between any type of biological entity such as proteins, genes, small molecules, metabolites, ligands, diseases, drugs, or even database records (Figure 6).

Some biological networks model the functions of cell- and tissue–specific molecular interactions at a cellular organizational level, varying from cells to a complete organ. These are:

### Protein-Protein Interaction Networks (PPIs)

This type of networks holds information about how different proteins operate with each other to enable a biological process within a cell. The interactions in a PPI network can be physical or predicted. Notably, a whole interactome can capture all PPIs happening in a cell or an organism. *In vivo* and *in vitro* methods for detecting PPIs include: X-ray crystallography, NMR, tandem affinity purification (TAP), affinity chromatography, coimmunoprecipitation, protein arrays, protein fragment complementation, phage display and yeast two-hybrid (Y2H) (Rao et al., 2014). Widely used repositories (Lehne and Schlitt, 2009; Szklarczyk and Jensen, 2015) which host PPIs for various organisms are the BioGRID (Stark et al., 2006), MINT (Chatr-aryamontri et al., 2007), BIND (Bader et al., 2003), DIP (Xenarios et al., 2000), IntAct (Hermjakob et al., 2004a), and HPRD (Peri et al., 2003) database. Concerning topology, the PPI networks follow a small-world property and are scale-free networks. Central hubs often represent evolutionarily conserved proteins, whereas cliques (fully connected subgraphs) have been found to have a high functional significance (Spirin and Mirny, 2003).

### Sequence Similarity Networks (SSNs)

These networks consist of nodes representing proteins or genes and edges capturing the sequence similarity between amino acid or nucleotide sequences. Widely used tools (Ekre and Mante, 2016) for obtaining a sequence similarity between two sequences are the BLAST (Altschul et al., 1990), LAST (Kiełbasa et al., 2011), and FASTA3 suite (Pearson, 2000), which contains SSEARCH, GGSEARCH, GLSEARCH executables of Smith-Waterman (Smith and Waterman, 1981) and Needleman-Wunsch (Needleman and Wunsch, 1970) implementations for local and global sequence alignment. These networks are weighted, have a small-world and scale-free topology and often contain hubs. Often, clustering algorithms are applied on such networks for the detection of protein families. Like in PPIs, proteins that lie together in such networks are more likely to have similar functions or be involved in similar biological processes (Sharan et al., 2007). While it is not straightforward to come to a conclusion about their density, when coping with fragmented sequences (e.g., alignments of predicted proteins from metagenomes), the networks are rather sparse.

### Gene Regulatory Networks

They are collections of regulatory relationships between transcription factors (TFs) and TF-binding sites or between genes and their regulators. Normally, these networks are directed, dynamic, and can be visualized as bipartite graphs. In such networks, most nodes have only a few interactions and only a few hubs come with a higher connectivity degree. In any case, such networks follow a power law degree distribution (scale-free) *p*(*k*) ~ *k*^−γ^, γ ≈ 2 (Vázquez et al., 2004). Among a variety of databases hosting information about gene regulation, widely used repositories are the KEGG (Kanehisa and Goto, 2000), GTRD (Yevshin et al., 2019), TRANSFAC (Matys et al., 2003), TRRUST (Han et al., 2018).

### Signal Transduction Networks

These networks capture cell signaling or otherwise the transmission of molecular signals as well as a series of molecular events within a cell or from the exterior to its interior (Fabregat et al., 2018). A signal transduction network normally consists of several thousand nodes and edges representing a series of reactions. These networks are mostly directed and sparse. They follow a power law degree distribution as well as small-world properties. While such data can be found in well-known pathway databases (KEGG, Reactome), specialized repositories such as the MiST (signal transduction in microbes) (Ulrich and Zhulin, 2007), NetPath (Kandasamy et al., 2010), or Human-gpDB (Satagopam et al., 2010) also exist.

### Metabolic Networks

They are networks consisting of metabolites (nodes) and their interactions in an organism. Metabolites can be either smaller molecules such as amino acids or larger macromolecules like polysaccharides. These networks are usually directed graphs and can be represented as Petri nets (Reisig, 1985; Chaouiya, 2007). They are scale-free, they carry small-world properties (Jeong et al., 2000) and can often be organized using hierarchies (Gagneur et al., 2003). In order to gain insights into their decomposition, heuristic modularity optimization over all possible divisions to find the best one is required (Newman and Girvan, 2004). KEGG and Reactome databases are two of the most widely used repositories for this type of network.

### Gene Co-expression Networks

They are undirected weighted networks where two nodes (genes) are connected if there is a significant co-expression between them. Such networks are usually constructed using data from high-throughput technologies such as Microarrays, RNA-Seq or scRNA-seq. For each pairwise connection, a metric like for example, the Pearson Correlation Coefficient (PCC) (Kirch, 2008) can be used to calculate an edge's weight. Often, a threshold or a *Z*-score are applied on the whole network in order to accept correlations above a certain cutoff. Otherwise the network would look like a fully connected clique. After the threshold and depending on the total clustering coefficient, the network can be clustered to detect functional modules. One typical example is the ribosomal genes which tend to group together due to similar expression patterns. Expression data for such analyses can be found in widely used repositories such as GEO (Barrett et al., 2013) or ArrayExpress (Parkinson et al., 2007). Notably, Arena-Idb (Bonnici et al., 2018) repository can be used for human non-coding RNAs interactions.

### Expression Quantitative Trait Loci (eQTL) Network

Data obtained from genotyping and/or transcriptomic experiments are used as locus (eQTLs) in explaining a fraction of the genetic variance of a gene expression phenotype (Nica and Dermitzakis, 2013). For this purpose, eQTL networks are suitable for summarizing this information (Platig et al., 2016; Fagny et al., 2017; Sonawane et al., 2019). Genome-wide association studies (GWASs) are used for association between common genetic variants and phenotypic traits based on many variants of relatively small effect size. Those single-nucleotide polymorphisms (SNPs) are measured by expression quantitative trait locus (eQTL) analysis and are represented by eQTL networks with significant associations as edges. Findings provide unique insight into the genotype–phenotype relationship [e.g., Enhanced tissue-specific heritability of type 2 diabetes (T2D) was identified by eQTL networks (Torres et al., 2014; Fagny et al., 2017)].

### lncRNA–Protein Interaction Networks

These networks reveal the functions of lncRNAs coming from their interactions with proteins (Yue et al., 2019). Most common experiments for studying these interactions include the *RNP immunoprecipitation-microarray (RIP-Chip)* (RIP-Chip: the isolation and identification of mRNAs, microRNAs, and protein components of ribonucleoprotein complexes from cell extracts), *high-throughput sequencing of RNA isolated by crosslinking immunoprecipitation (HITS-CLIP)* (HITS-CLIP yields genome-wide insights into brain alternative RNA processing*.), photoactivatable ribonucleoside-enhanced crosslinking and immunoprecipitation (PAR-CLIP)* (Transcriptome-wide identification of RNA-binding protein and microRNA target sites by PAR-CLIP.) and *RNAcompete* (Rapid and systematic analysis of the RNA recognition specificities of RNA-binding proteins). Regarding the computational methods for predicting these interactions, network-based methods are the most applicable*. Multiple protein–protein similarity networks (PPSNs)* (Fusing multiple protein-protein similarity networks to effectively predict lncRNA-protein interactions.), *LPIHN* (Predicting Long Noncoding RNA and Protein Interactions Using Heterogeneous Network Model) and *PLPIHS* (Prediction of lncRNA-protein interactions using HeteSim scores based on heterogeneous networks) could be applied to generate lncRNA–protein interaction networks (Zhang et al., 2019).

Additionally, some biological networks are distinguished by comprising information about evolution and interactions of species. These are:

**Phylogenetic networks:** They are networks trying to capture the evolutionary relationships between organisms in time (Huson et al., 2010; Thomas and Portier, 2013). Reconstructed phylogenies are mainly represented as trees even if it is debatable whether a tree is the right scheme as it fails at capturing events like the union of different lineages. As an extension to trees, phylogenetic networks might contain loops. The tree of life is a global effort to capture the evolution of all organisms in a single snapshot and describe the relationships between them. Notably, widely used methods for tree reconstruction are the *Neighbor-Joining (NJ)* (Saitou and Nei, 1987), *UPGMA*, and *maximum likelihood parsimony* (Golding and Felsenstein, 1990), whereas widely used applications for such analyses are the PAUP (Yang, 1996), PHYLIP (Baum, 1989), and MEGA (Kumar et al., 2016).
**Ecological networks:** These networks mainly represent food webs or interactions among species in an ecosystem. These interactions can be trophic or symbiotic (Ings et al., 2009), mutualistic (bidirectional) or competitive (host-parasite). A fundamental aim of ecological network analysis is to uncover the mechanisms which influence the stability of fragile ecosystems. In general, binary food webs can be simple directed or undirected *k-*partite or simple graphs, whereas quantitative food chains can be shown as weighted graphs. Most food webs follow an exponential degree distribution, whereas it is well-accepted that such webs display an average low connectance. An in-depth analysis of the topological features of this type of networks is extensively discussed elsewhere (Danon et al., 2011).
**Epidemiological networks:** They are networks used in public health to study disease transmission (e.g., sexually transmitted diseases—STDs) (Danon et al., 2011). Path traversal analysis can reveal transmission routes while the network's structure can provide insights into the epidemiological dynamics. While epidemiological networks often simulate social networks, they can be shown as bipartite graphs (Pavlopoulos et al., 2018).
**Species interaction networks:** There are *between-species interaction networks* describing pairwise interactions between species, trying to understand what factors (e.g., diversity) lead to stability (Romanuk et al., 2010) and *within-species interaction networks* quantifying associations between individuals, offering information in species, and/or population level (Croft et al., 2004).
**Food webs:** All organisms are connected to each other through feeding interactions and the networks presenting these interactions are very—well known for the effort to answer the long-standing question in ecology about the stability of these interactions (Milner-Gulland, 2012).
Interactions of ecological entities captured in networks can be obtained from literature articles, observation in the field, molecular experiments (e.g., analysis of environmental DNA), or models based on incomplete data (Delmas et al., 2019).

Moreover, *biomedical graphs* are of great importance for both researchers and clinicians (Yue et al., 2019). These are:

**Disease networks:** They are formed by diseases and their causative genes, while the connections between them can be constructed based on repositories such as the Online Mendelian Inheritance in Man (OMIM) associations. These networks are generated when diseases share at least one causative gene, and therefore are considered to be linked. Disease networks are typically shown as bipartite networks (Goh et al., 2007).
**Drug-disease associations:** These networks hold information about known and/or predicted drug-disease associations. The information could be extracted from a database or from published literature (Gottlieb et al., 2011; Sonawane et al., 2019; Yue et al., 2019).
**Disease—symptom graphs:** These graphs connect diseases with their symptoms and visualize the potential evolution of the diseases, assisting clinicians to follow the more efficient medical treatment rapidly (Sonawane et al., 2019). These graphs are generated based on medical records using rudimentary concept extraction of cause and effect.

Finally, data integration approaches can be used to generate biological networks consisting of nodes which represent text, database records, or literature articles.

**Literature co-occurrence networks:** These networks show connections between bioentities that are found to co-occur in any text corpus (Pavlopoulos et al., 2014). Name-entity-recognition (NER) taggers such as the EXTRACT (Pafilis et al., 2016), can be used to initially identify genes/proteins, chemical compounds, environments, tissues, diseases, phenotypes, and Gene Ontology terms in a text and map the identified terms to their corresponding ontology/taxonomy entries in public databases. This way, any text corpus like Wikipedia, PubMed (~29 million abstracts) or PubMed Central (PMC, ~6 million full-text articles) can be parsed and analyzed for both abstract-based or sentence-based co-occurrences.
**Knowledge networks:** These networks are mostly multi-edge graphs as they combine heterogeneous information and metadata from various sources like public repositories or biological and literature databases. Typical examples are the STRING (Franceschini et al., 2013), STITCH (Szklarczyk et al., 2016), and PICKLE (Gioutlakis et al., 2017) databases. STRING contains known and predicted protein-protein interactions for various organisms, whereas STITCH contains known and predicted interactions between chemical compounds and proteins. In the STRING database, two proteins can be, for example, connected in multiple ways. They can be homologous, or co-occur in an abstract, or have neighboring positions in a genome or be products of a fusion event or co-express in an experiment. Similarly, PICKLE integrates publicly available PPI databases via genetic information ontology. Finally, bioDBnet (Mudunuri et al., 2009) is a network of the major biological databases.

Overall, biological networks follow the new era of *hybrid heterogeneous networks*, trying to put together different types of information (Navlakha and Kingsford, 2010; Moreau and Tranchevent, 2012; Ni et al., 2016). It is worth mentioning, that a great collection of biological networks that are produced by researchers and are published in various articles can be found in https://cytoscape-publications.tumblr.com. This repository can be used as an excellent teaching material as well as a great resource for inspiration and case studies when building software applications.

## Functional Annotation and Overrepresentation Analysis

A common task in computational biology field is the annotation and interpretation of gene lists (e.g., genes or proteins which are found to be tightly connected in a network). For this task, functional annotation and/or overrepresentation analysis can be used (Tipney and Hunter, 2010; Hung et al., 2012). Enrichment analysis determines over-represented classes of genes or proteins in a large group of samples in order to reveal existing associations with disease phenotypes (Huang et al., 2009a). Similarly, functional enrichment analysis applies statistical tests to match genes of interest with certain biological functions (Bindea et al., 2009). PANTHER (Mi et al., 2013), Gorilla (Eden et al., 2009) and DAVID (Huang et al., 2009b) applications for example, accept a gene list as an input and report related hits to molecular functions, biological processes [e.g., Gene Ontology (Gene Ontology Consortium, 2004)] and KEGG (Kanehisa and Goto, 2000) and Reactome (Fabregat et al., 2018) pathways. Another similar tool is the ClueGO (Bindea et al., 2009) which is offered as a Cytoscape plugin. For researchers interested in non-coding RNA annotation and identification, Transcriptator (Tripathi et al., 2015) can be used. Pathway enrichment analysis can be also performed by additional tools such as pathfindR (Ulgen et al., 2019), g:Profiler (Raudvere et al., 2019), and EnrichmentMap (Merico et al., 2010; Reimand et al., 2019). Gene Set Enrichment Analysis [GSEA (Mootha et al., 2003; Subramanian et al., 2005)] and NGSEA (Han et al., 2019) can be used for overrepresentation analysis, whereas differential expression analysis for the determination of the up- and down- regulated genes is offered by DESeq2 (Michael, 2017) or metaseqR (Moulos and Hatzis, 2015).

## File Formats

**Figure 7:**
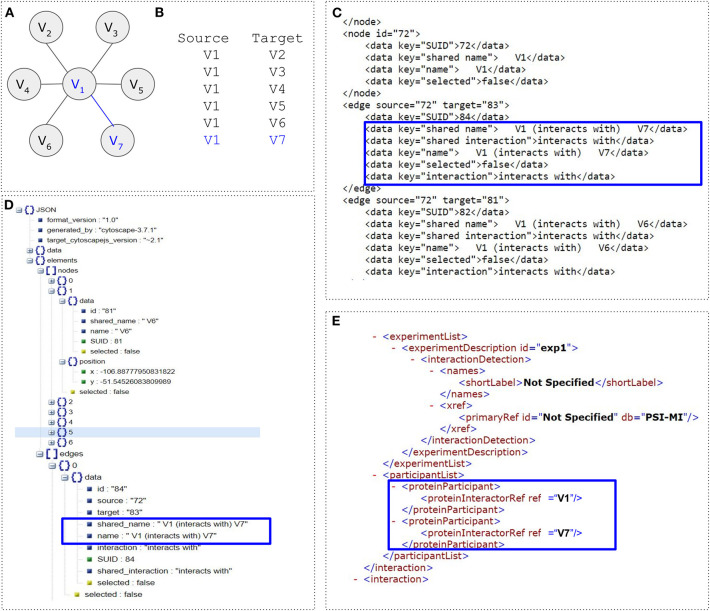
Examples of file formats. **(A)** Simple undirected graph consisting of seven nodes (*V* = 7) and six edges (*E* = 6). **(B)** Network in Tab-delimited file format. **(C)** Network in GraphML file format. Blue box highlights the interaction between nodes *V*_1_ and *V*_7_. **(D)** A cytoscape.js graph encoded in JSON. **(E)** Network in PSI-MI file format.

A network can be described and stored in multiple human- and computer-readable ways. Apart from the simple file formats such as the tab-delimited, CSV, SIF, Excel and adjacency matrix, several others like the BioPAX (Demir et al., 2010), SBML (Hucka et al., 2003), PSI-MI (Hermjakob et al., 2004b), CML (Murray-Rust et al., 2001), and CellML (Lloyd et al., 2004) have been introduced for biological data and semantics. For example, SBML, which stands for Systems Biology Markup Language, is an XML-like format for storing and parsing biochemical networks as well as for describing biological processes. BioPAX stands for Biological Pathway Exchange and is made for the representation of biological pathways at the molecular and cellular level. The PSI-MI format is used for the data exchange related to molecular interactions and CellML is used for describing mathematical models. GraphML (Brandes et al., 2017) is an XML-like file format and consists of unordered sections related to a network's node and edge elements. Each node has a distinct identifier, whereas each edge is described by a source and a target node. Additional attributes such, an edge weight or a label can also be included in the schema. The JavaScript Object Notation (JSON) format is a generic and widely-used non-biological file format and is popular for web–based applications or web-server asynchronous communication and data exchange. However, it is worth mentioning that Cytoscape.js (Franz et al., 2016) accepts JSON formats for network visualization. Finally, the Nexus and the Newick file formats are standard ways for representing trees. While NDEx (Pillich et al., 2017) is an open-source framework for the sharing of networks of many types and formats, file-format-specific parsers are available [e.g., Bioconductor (Gentleman et al., 2004) rBiopaxParser (Kramer et al., 2013), rsbml, RPsiXML and others]. Examples of such file formats are shown in Figure 7.

## Graph Layouts and Edge Bundling

For graph analysis and interpretation, it is important to be able to depict a graph whose structure, symmetries, and other main features become clear in a visually and aesthetically appealing way. This is especially true for graphs of large size, where many nodes and edges can have multiple clusters and interconnected areas.

**Figure 8:**
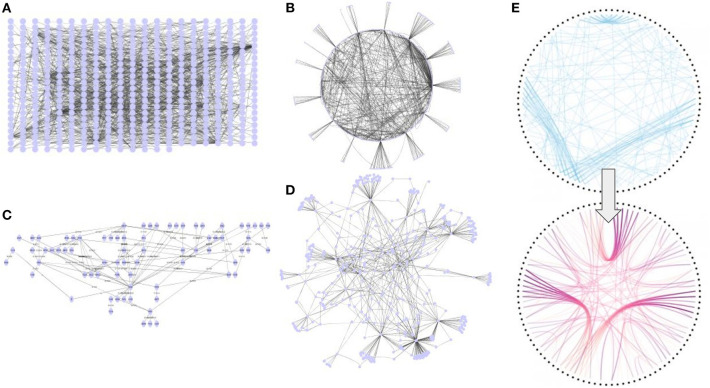
Network layouts. **(A)** Grid layout. **(B)** Circular layout. **(C)** Hierarchical layout. **(D)** Force-directed layout **(E)** Edge-bundling. All views have been generated with Cytoscape.

Graph drawing combines methods from mathematics and computer science to derive two- and three- dimensional representations of graphs, employing a number of strategies (Figure 8). Among the most successful layouts are the ***force-based layout*** approaches, where the nodes of the graphs are metaphorically modeled as point particles with attractive (spring) forces acting between nodes connected by an edge and repelling (electrical) forces acting between all pairs of nodes. The optimal layout is determined by the positions of nodes/particles that minimize the total energy of the system. Typically, such a state is found by simulating the forces of the many-particle physical system and arriving at a minimum energy state iteratively. In addition, in a ***spectral layout*** method, the coordinates are taken to be the eigenvectors of a matrix such as the Laplacian, derived from the adjacency matrix of the graph. ***Orthogonal layout*** methods allow the edges of the graph to run horizontally or vertically, parallel to the coordinate axes of the layout, while ***tree layout*** algorithms use tree-like structures and are suitable for visualizing ontologies or hierarchies. Finally, ***circular layout*** methods place the vertices of the graph on a circle, choosing carefully the ordering of the vertices around the circle to reduce crossings and place adjacent vertices close to each other.

While graph drawing is a mature field with many proposed alternatives, the approaches that produce the most compelling visualizations (e.g., force directed based algorithms) can often become CPU and memory greedy and struggle with visualizing networks of more than a few thousands of nodes and edges. Empirical performance statistics can be found elsewhere (Pavlopoulos et al., 2017). Many layout algorithms are embedded in standard visualization tools: Gephi (Bastian et al., 2009) visualization tool comes with a great variety of algorithms such as *OpenOrd* (Martin et al., 2011) and *Yifan-Hu* (Yifan, 2005) force-directed algorithms. OpenOrd can layout networks consisting of over a million nodes in less than half an hour but aesthetics depends on the network's topology. The Yifan-Hu layout can give aesthetically comparable representations to the ones produced by the widely used but time-consuming *Fruchterman-Reingold* (Fruchterman and Reingold, 1991), with much faster performance. Other algorithms included in Gephi are the circular, contraction, dual circle, random, MDS, Geo, Isometric, GraphViz, and Force atlas layouts. Similarly, Cytoscape (Shannon et al., 2003) visualization tool comes with a rich variety of simple (grid, random, and circular) and more sophisticated (force-directed, hierarchical) layout algorithms. Finally, for more customized layouts, one can utilize the igraph library (Gabor and Nepusz, 2006). yWorks provides the professional software manufacturer with state-of-the-art diagramming components.

For even more aesthetic layouts, edge bundling methods can be utilized to provide significant clutter reduction and make visible high-level edge patterns clearer (Zhou et al., 2013). These methods are gaining ground over the years and are mostly divided in hierarchical or force directed. An overview of these methods is extensively described elsewhere (Zhou, 2016). Edge bundling methods are still computationally expensive and their main philosophy is to group edges together like a bundle of cables. Cytoscape and Tulip (Auber et al., 2017) are two of the most widely used visualization tools which have such methods incorporated. Basic node layout as well as edge-bundling examples are shown in Figure 8.

In general, force-directed layouts are very suitable for scale-free networks like PPIs or highly modular networks with distinct communities or high clustering coefficient. It would not make sense for example to apply a force-directed layout in a fully connected graph. Similarly, hierarchical layouts are more suitable for trees or tree-like graphs such as the Gene Ontology. Finally, it is worth mentioning that there is tradeoff against time, particularly because algorithms (e.g., layouts) grow time exponentially as the network increases.

## Network Visualization

Several techniques have been introduced for the visualization of networks varying from very simple (e.g., *adjacency matrices*) to more complex (e.g., *force directed layouts* in 2D or 3D). However, the selection of the appropriate visualization, highly depends on the type of network which needs to be visualized. For example, in a multi-Omics approach, one would like to see different types of information (e.g., proteomics, transcriptomics, metabolomics, genomics) in a well-structured view. For this purpose, a *multi-layered visualization* would be much more preferable compared to a generic force directed layout. This way, nodes of different type are placed onto different layers, while connections are allowed both within a layer as well as across layers. In the case of *multi-edge graphs*, two bioentities can be connected in multiple ways. Two genes, for example, might be homologous, or neighbors in a genome or co-express in an experiment. STRING database is one of the most widely used databases which utilizes multi-edge graph visualization. In such networks, layouts can be applied taking into consideration only one connection type or any combination of them.

**Figure 9:**
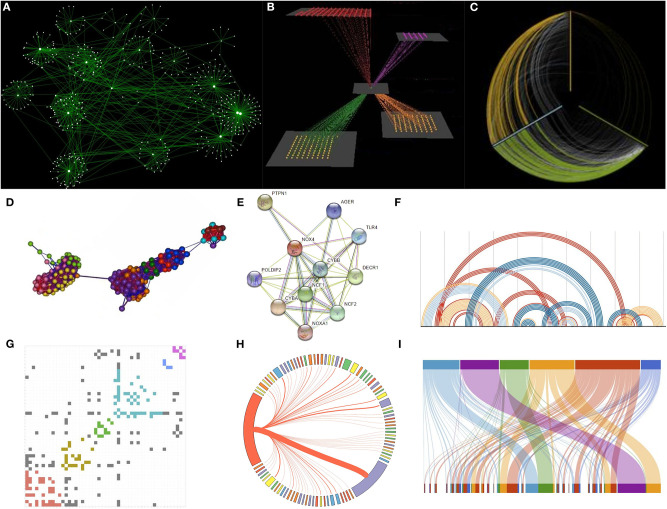
Network representations. **(A)** A network visualized by Cytoscape with the use of a force-directed layout algorithm. **(B)** A multi-layered graph visualized by Arena3D. **(C)** A hive-plot view. **(D)** A network in 3D visualized by Graphia application **(E)** A multi-edge network visualized by STRING. **(F)** A network visualized with the use of arcs. **(G)** A network visualized as a colored adjacency matrix. **(H)** A circular Circos view. **(I)** Visualization of a bipartite graph.

While force-directed or hierarchical visualizations are very common, they often fail in coping the so-called *hairball effect* (dense networks where all nodes are almost connected to any other node—no structure). To partially address this issue, *circos* and *hive plots* have been introduced. *Hive plot* views use “radially oriented” linear axes as a coordinate system. Nodes are placed on these axes and edges are drawn as curved links. While hive plots are general, they have been used in biology to successfully visualize cancer, gene-disease, and gene regulatory networks (Krzywinski et al., 2012). Similarly, Circos application (Krzywinski et al., 2009) enables a *circular composition* to show connections between nodes or positions, which are difficult to visually organize when the underlying layout is linear. Such plots are very widely used in biology to represent phenomena like genomic variations. *Arc diagrams* in which nodes are displayed along a single axis and links are represented with arcs, can be used for a similar purpose. Finally, *bipartite graphs* which are widely used in epidemiology and gene-disease networks need special visualization to show mutual relationships between the elements of their two collections. While several other visualization approaches can be applied on hierarchical graphs (e.g., Gene Ontology) and biochemical networks (e.g., pathways or petri nets), the most basic concepts are schematically shown in Figure 9.

## Graph-Based Clustering

Clustering is the process of grouping a set of objects so that objects belonging in the same group (cluster) have similar properties. For this, many state-of-the-art algorithms take into account the network's topology and try to cluster the network accordingly. For example, many approaches try to find densely connected areas in a network, others try to “break” the bridges (edges with high betweenness centrality) between distinct communities and others look for easiest flow paths or are based on node distances.

Despite the great variety of graph-based clustering algorithms available today (Xu and Wunsch, 2005; Brohée and van Helden, 2006; Moschopoulos et al., 2011), only few can cope with large-scale networks consisting of millions of nodes and edges. SPICi (Jiang and Singh, 2010) is one of the fastest algorithms and accepts as input a list of connections. It supports both dense and sparse matrices and tries to find local densely connected neighbors using heuristics. It has running time complexity *O(VlogV*+*E)* time and needs *O(E)* memory. It is not suitable for networks with many hubs and low clustering coefficient. Louvain (Blondel et al., 2008) on the other hand, is an old-fashioned but rather fast and greedy algorithm with *O(VlogV)* time performance. Molecular Complex Detection (MCODE) (Bader and Hogue, 2003) is a widely-used algorithm in biology and very suitable for finding protein complexes in PPI networks. It has *O(VEd*^*3*^*)* time complexity where *d* is the vertex size of the average vertex neighborhood in the input graph. Affinity-propagation (Frey and Dueck, 2007) detects ways that nodes in a network can exchange “messages” between each other very fast. It is a high-quality algorithm and comes with *O(V*^2^) time complexity. This might be a decent performance for medium-scale biological networks like gene co-expression or PPIs but not sufficient for larger networks like the literature-based or the knowledge-based ones. Markov Clustering (MCL) (Enright et al., 2002) is one of the mostly cited algorithms in the field and was initially introduced to detect protein families from sequence similarity networks. It uses random walks to detect highly-connected subgraphs using a mathematical bootstrapping procedure and is able to cluster a few million nodes in less than an hour. However, it is memory greedy, a bottleneck which has been solved with its parallel version HipMCL (Azad et al., 2018), a scalable distributed-memory implementation. HipMCL uses MPI (Forum, 1994), and OpenMP (Dagum and Menon, 1998) and can cluster a network consisting of 300 million nodes and ~17 billion edges in only ~6 h using ~136,000 cores.

**Figure 10:**
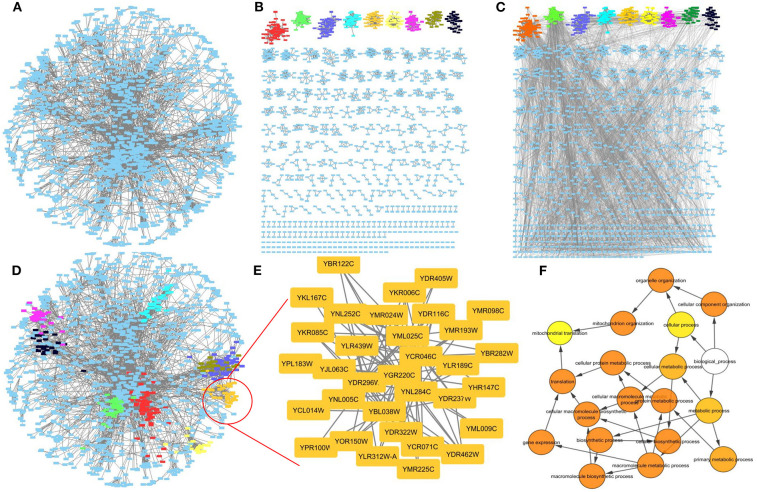
Network clustering. **(A)** A Yeast PPI network. **(B)** The PPI network clustered with MCL. **(C)** The PPI network clustered with MCL with the initial connections restored. **(D)** The initial network structure with some MCL clusters highlighted. **(E)** A cluster in high resolution. **(F)** Gene Ontology enrichment related for the zoomed cluster. Visualization is offered through Cytoscape whereas clustering has been performed with the use of ClusterMaker2 plugin.

While it is not in the scope of this review to go into each algorithm's detail, we highly encourage readers to either try each of them individually in their command line versions or through the clusterMaker2 (Morris et al., 2011) Cytoscape plugin. In Figure 10 for example, a Yeast PPI network (Gavin et al., 2006) has been clustered with clusterMaker's MCL algorithm, whereas a randomly selected cluster has been annotated with Cytoscape's BiNGO plugin (Maere et al., 2005).

## Hierarchical Clustering

Hierarchical clustering is a non-graph-based way of data clustering which accepts a distance matrix containing all pairwise distances between the nodes as input and outputs a dendrogram showing the hierarchical relationship between the clusters. The standard hierarchical algorithm has *O*(*n*^3^) time complexity of and requires *O*(*n*^2^) memory, thus making this method inappropriate for large data sets. Hierarchical clustering is divided in three main categories. These are *Single linkage* which calculates the smallest distance between objects in each iteration step, *Complete linkage* which calculates the longest distance between objects in each iteration step and *Average linkage* which uses the average distance between all pairs of objects in every iteration step. For more details, a survey explaining how hierarchical clustering algorithms work and what are their variations can be found elsewhere (Langfelder et al., 2008).

**Figure 11:**
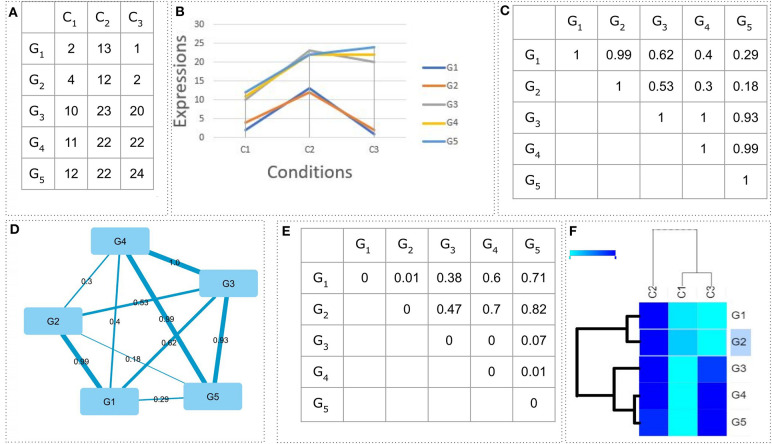
Example of hierarchical clustering. **(A)** The expression values of five genes in three conditions. **(B)** The chart showing the genes' expression values as patterns. **(C)** The Pearson correlation coefficient (PCC) matrix showing all pairwise PCC values. **(D)** The Pearson correlation matrix in the form of a fully connected graph. **(E)** The distance matrix as a product of the PCC matrix (*D*_*ij*_ = 1 − *PCC*_*ij*_). **(F)** A 2D average linkage hierarchical clustering. Genes *G1, G2* as well as genes *G3, G4, G5* are clustered together.

Notably, all calculations are based on a distance matrix (fully connected graph) which can be generated by a correlation matrix as *D*_*ij*_ = 1 − *PCC*_*ij*_. *D* is the distance matrix and *PCC* a Pearson Correlation Matrix (e.g., gene co-expression networks). Figure 11 shows an example of how five genes can be hierarchically clustered according to their expression values/patterns measured in three hypothetical conditions or time points. The final output is a heatmap accompanied by a dendrogram showing how genes are grouped together. Notably, in cases where it is not straightforward which cutoff to apply on the tree in order to define the number of clusters, statistical methods to automate such task, are available (Langfelder et al., 2008).

## Clustering Comparison

Different clustering algorithms or runs of the same algorithm using different parameters can often lead to dissimilar results. Therefore, it is essential to be able to compare different clustering results between each other. This is especially useful when one wants to compare the results of a clustering algorithm against an “optimal” or desired clustering for example, to study an algorithm's accuracy.

For this purpose, several clustering comparison metrics have been introduced. Generally speaking, these metrics can be divided into three categories: (i) counting pairs, (ii) set overlaps, or (iii) mutual information (Wagner and Wagner, 2007). Some well-known clustering comparison metrics which are based on counting pairs are the *Chi Squared Coefficient* (Mirkin, 2001), *Rand Index* (Rand, 1971), *Fowlkes–Mallows Index* (Fowlkes and Mallows, 1983), *Mirkin Metric* (or *Equivalence Mismatch Distance*), *Jaccard Index* and the *Partition Difference* (Li et al., 2004). Metrics based on set overlaps include the *F-Measure* (Fung et al., 2003), *Meila-Heckerman & Maximum-Match-Measure* (Marina Meil and David, 2001), and the *Van Dongen-Measure* (Dongen, 2000). Finally, clustering comparison metrics of the mutual information category include the *Normalized Mutual Information by Strehl & Ghosh* (Alexander and Joydeep, 2003), *Normalized Mutual Information by Fred & Jain* (Ana and Jain, 2003) and the *Variation of Information* methods (Meila, 2000).

The metrics of the first category count the number of object pairs that (a) were clustered together in both clusterings, (b) were clustered differently in both clusterings and (c) were clustered together in only one of the two clusterings. *Rand Index* is such a metric, ranging from 0 to 1 and is defined as: $RandIndex\left(C_{1},C_{2}\right)\;=\;\frac{2\left(n_{ctb}+n_{cdb}\right)}{n\left(n-1\right)}$ where *C*_1_ and *C*_2_ are two different clusterings of a data set with *n* objects, *n*_*ctb*_ is the total number of object pairs that were clustered together in both clustering and *n*_*cdb*_ the number of pairs that were clustered differently in both clusterings. Intuitively, the *Rand Index* calculates the fraction of same-clustered (together or separately) pairs against the number of all possible pairs and equals to 1 when all pairs are clustered in the same manner in both clusterings and to 0 when there is no pair clustered in the same manner in any of the two clusterings.

Metrics based on set overlaps try to map clusters between clusterings in accordance to their maximum overlap. The *Meila-Heckerman* measure compares the results of a clustering against the optimal clustering. This makes the method asymmetric, which means it cannot be used while comparing two clusterings without one being the optimal. The *Maximum-Match-Measure* is the symmetric version of this metric which iteratively looks for the largest element of the confusion matrix of the intersection values between all clusters of the two clusterings, meaning the cluster pair with the largest overlap. The column and row of the confusion matrix which contain the largest element are then crossed out and the sum of the results of all iterations are aggregated and divided by the total number of elements. The formula for the *Maximum-Match-Measure* is as follows: $MM\left(C_{1},C_{2}\right)=\frac{1}{n}\overset{min\left\{k,l\right\}}{\underset{i\;=\;1}{\sum }}max\left\{conf^{\prime }\right\}\;$where the algorithm finishes in *min*{*k, l*} steps, *k* and *l* are the respective numbers of clusters for clusterings *C*_1_ and *C*_2_ and *conf*′ is the confusion matrix described above with *i* − 1 columns and *i* − 1 rows being removed at each iteration. *Maximum-Match-Measure* ranges from 0 to 1.

An asymmetric and widely used clustering comparison metric of the set-overlap category is the *F-Measure*. The *F-Measure* indicates how close a clustering *C*_2_ is to an optimal clustering *C*_1_ by making use of the harmonic mean of precision and recall between each cluster, with precision $pC_{1i}C_{2j}=\frac{conf_{ij}}{nC_{2j}}$ and recall $rC_{1i}C_{2j}=\frac{conf_{ij}}{nC_{1i}}$, *i ϵ* [1, *k*] and *j ϵ* [1, *l*]. The *F-Measure* between two clusters is calculated as *F*(*C*_1*i*_, *C*_2*j*_) = $\frac{2\;*\;pC_{1i}C_{2j}\;*\;rC_{1i}C_{2j}}{\;pC_{1i}C_{2j}\;+\;rC_{1i}C_{2j}}$ and the overall *F-Measure* between two clusterings is defined as the weighted sum of the maximum *F-Measures* for the clusters in *C*_2_, $F\left(C_{1},C_{2}\right)=\overset{k}{\underset{i\;=\;1}{\sum }}\frac{nC_{1i}}{n}max_{j=1}^{l}\left\{F\left(C_{1i},C_{2j}\right)\right\}$ and ranges in [0, 1].

Metrics of the mutual information clustering comparison category are based on the entropy of information and on the probability of finding an element in a specific cluster. The entropy of a clustering is defined as $H\left(C\right)\;=\;-\overset{k}{\underset{i\;=\;1}{\sum }}P\left(i\right)log_{2}p\left(i\right),\;$ where $P\left(i\right)=\frac{nC_{i}}{n}$ is the probability that a random element picked is a member of cluster *C*_*i*_, and the mutual information between two clusterings *C*_1_ and *C*_2_ as $I\left(C_{1},C_{2}\right)=\overset{k}{\underset{i\;=\;1}{\sum }}\overset{l}{\underset{j\;=\;1}{\sum }}P\left(i,j\right)log_{2}\frac{P\left(i,j\right)}{PC_{1}\left(i\right)PC_{2}\left(j\right)}$, where $P\left(i,j\right)\;=\;\frac{{conf}_{ij}}{n}$ is the probability that an element belongs in cluster *C*_1*i*_ and also in *C*_2*j*_. The *Variation of Information* is a mutual information clustering comparison metric and is calculated as *VI*(*C*_1_, *C*_2_) = *H*(*C*_1_) + *H*(*C*_2_) − 2*I*(*C*_1_, *C*_2_). Intuitively, the *Variation of Information* metric describes the amount of information we lose from the first clustering as well as the information we still have to gain from the second clustering. The *Variation of Information* metric is not bounded by a constant value but by a *log*(*n*)upper bound (in the case of two trivial clusterings).

**Figure 12:**
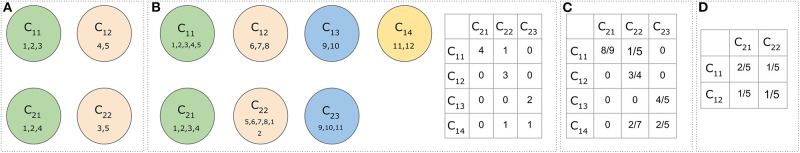
Clustering comparisons. **(A)** Rand Index between *C*_1_ and *C*_2_. *C*_11_ and *C*_12_ are clusters 1 and 2 of the *C*_1_ clustering, respectively. One pair [1, 2] is clustered together in both clusterings, three pairs [1, 5], [2, 5], and [3, 4] are clustered differently in both clusterings and the rest six pairs [1, 3], [1, 4], [2, 3], [2, 4], [3, 4], and [4, 5] have been placed together in only one of the two clusterings. The Rand Index between the two clusterings is calculated as $RandIndex\left(C_{1},C_{2}\right)\;=\;\frac{2\left(1+3\right)}{5\left(4\right)}=0.4$. **(B)** Maximum-Match-Measure between *C*_1_ and *C*_2_. *C*_1_ has four clusters while *C*_2_ has three. At the first iteration the cluster-intersections' confusion matrix element $con{f^{\prime }}_{\;11}=4$ is chosen and column 1 and row 1 are crossed out. At the second iteration the maximum element of the remaining confusion matrix is $con{f^{\prime }}_{\;22}=3$ and column 2 and row 2 are crossed out. At the third and final iteration $con{f^{\prime }}_{\;33}=2$ is chosen. The metric is calculated as: $MM\left(C_{1},C_{2}\right)=\frac{1}{12}\overset{3}{\underset{i\;=\;1}{\sum }}max\left\{conf^{\prime }\right\}=\frac{1}{12}\left(4+3+2\right)=0.75$. On the same schema if *C*_1_ is chosen as the optimal clustering the *F-measure* for *C*_2_ can be calculated. First, the precision and recall measures are calculated for clusters *C*_11_ and *C*_21_ as $pC_{11}C_{21}=\frac{conf_{11}}{nC_{21}}=\frac{4}{4}=1$ and $rC_{11}C_{21}=\frac{conf_{11}}{nC_{11}}=\frac{4}{5}$. Then, the *F-Measure* can be calculated between these two clusters as *F*(*C*_11_, *C*_21_) = $\frac{2\;*\;pC_{11}C_{21}\;*\;rC_{11}C_{21}}{\;pC_{11}C_{21}\;+\;rC_{11}C_{21}}$ = $\frac{2\;*1\;*\frac{4}{5}}{1\;+\;\frac{4}{5}}$ = $\frac{8}{9}$. By calculating the respective values for the rest of the cluster pairs, the matrix **(C)** is created. The overall F-Measure of *C*_2_ against *C*_1_ is $F\left(C_{1},C_{2}\right)=\overset{k}{\underset{i\;=\;1}{\sum }}\frac{nC_{1i}}{n}max_{j=1}^{l}\left\{F\left(C_{1i},C_{2j}\right)\right\}=\frac{5}{12}*\frac{8}{9}+\frac{3}{12}*\frac{3}{4}+\frac{2}{12}*\frac{4}{5}+\frac{2}{12}*\frac{2}{5}≃0.76$. **(D)** *Variation of Information* matrix of the *P*(*i, j*) probabilities of an element being in the intersection of clusters. Based on the two clustering schemas of **(A)** the entropy of *C*_1_ is *H*(*C*_1_) = $-\overset{2}{\underset{i\;=\;1}{\sum }}P\left(i\right)log_{2}p\left(i\right)$ = $-\left(\frac{3}{5}log_{2}\left(\frac{3}{5}\right)\;+\frac{2}{5}log_{2}\left(\frac{2}{5}\right)\right)≃0.97$ and following the same procedure *H*(*C*_2_) ≃ 0.97. The mutual information between the two clusterings is calculated as *I*(*C*_1_, *C*_2_) = $\overset{k}{\underset{i\;=\;1}{\sum }}\overset{l}{\underset{j\;=\;1}{\sum }}P\left(i,j\right)log_{2}\frac{P\left(i,j\right)}{PC_{1}\left(i\right)PC_{2}\left(j\right)}=\frac{2}{5}log_{2}\left(\frac{\frac{2}{5}}{\frac{3}{5}*\frac{3}{5}}\right)\;+\frac{1}{5}log_{2}\left(\frac{\frac{1}{5}}{\frac{3}{5}*\frac{2}{5}}\right)\;+\;\frac{1}{5}log_{2}\left(\frac{\frac{1}{5}}{\frac{2}{5}*\frac{3}{5}}\right)\;+\;\frac{1}{5}log_{2}\left(\frac{\frac{1}{5}}{\frac{2}{5}*\frac{2}{5}}\right)≃0.02.\;$The final value of the *Variation of Information* metric becomes *VI*(*C*_1_, *C*_2_) = *H*(*C*_1_) + *H*(*C*_2_) − 2*I*(*C*_1_, *C*_2_) = 0.97 + 0.97 − 2 * 0.02 = 1.9.

Examples of *Rand Index, Maximum-Match-Measure, F-Measure* and *Variation of Information* are shown in Figure 12.

## Network Alignment

In today's multi-Omics era, integration of heterogeneous information (e.g., transcriptomics, proteomics, metabolomics, etc.) in a multi-layered network structure is becoming a trend. Additionally, methods to directly compare networks and their topological features are gaining ground. To address these issues, network alignment, or alternatively graph isomorphism approaches can be used. Notably, graph alignment is not a trivial task as it is computationally expensive and has been characterized as *NP*-complete (Zampelli et al., 2010). The concept behind network alignment is to highlight conserved or missing nodes and edges across two (pairwise) or more (multiple) networks. In the biomedical field for example, an alignment could potentially be used for the discovery of conserved traits between different species (Sharan et al., 2005), the detection of common pathway interactions between two different disease states or the detection of deleted gene expression connections upon drug treatment. Like in a sequence alignment, a network alignment can also be either *local* or *global*.

Established implementations in the field include the NetworkBLAST aligner (Kalaev et al., 2008) for protein network alignment between two species or across multiple networks from different organisms, the MaWISh (Maximum Weight Induced Subgraph) (Koyutürk et al., 2006) for PPI alignments in order to underlie evolutionary relationships and the H-GRAAL (Milenković et al., 2010) for metabolic networks of different species.

Until now, several graph alignment strategies have been introduced and various methods have been implemented. Some of the strategies are: *modular graph kernels* and *divide and conquer* strategies (Towfic et al., 2009), *constraint programming* (Zampelli et al., 2010), *linear representation of networks* (Kalaev et al., 2008), *scoring functions* (Flannick et al., 2008), *connected-components* (Tian and Samatova, 2008), *heuristic searches* (Kuchaiev et al., 2010), and *graphlet degree vectors*. Notably, recent developments allow the alignment of networks with multiple edge types (Gu et al., 2018).

**Figure 13:**
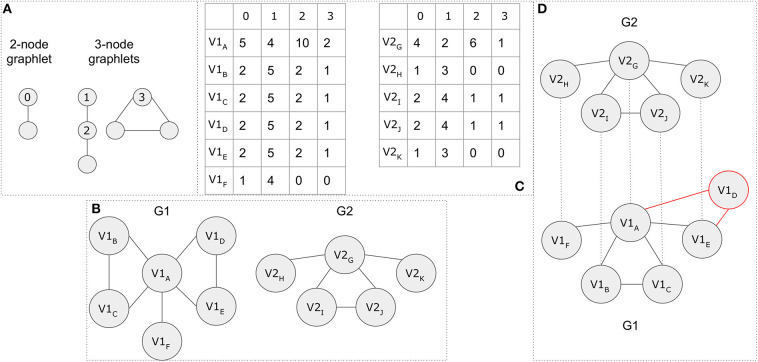
A topology-based network alignment example. **(A)** Possible graphlet compositions for 2 and 3 nodes. Orbits 0–3, which are annotated, represent the possible position for a node in the various graphlets. **(B)** *G1* and *G2* graph representations. **(C)** Graphlet degree signatures. The row names represent the nodes, while the column names the different orbits. **(D)** The final network alignment based on a simplified version of the GRAAL algorithm. Node *V1*_*D*_ (in red color) remains unaligned.

While it is not in the scope of this article to cover all existing methods, for demonstration purposes, we present an example based on a simplified version of the GRAAL alignment method. The GRAAL network aligner takes into consideration the topology of a network and uses facets from both local and global alignment methods to produce a global alignment. According to the GRAAL algorithm, each node of the smaller network is aligned to exactly one node of the larger one. Let's assume that there are two graphs *G*1 = (*V*1, *E*1) and *G*2 = (*V*2, *E*2). GRAAL introduces the concept of graphlets, which give a node a more detailed representation of its degree based on its local neighborhood of connections. All possible 2- and 3-node graphlet compositions are shown in Figure 13A. The aggregation of the number of 2-node graphlets attached to a node, represents the node's degree. A node can only appear in one of the annotated orbits 0–3 in the respective graphlets in Figure 13A.

Figure 13B, demonstrates a network alignment example. During GRAAL's first step, the lowest possible alignment cost for aligning each node from *G1* to each node of *G2* is calculated. Each row in Figure 13C represents the graphlet-signature for each node (for each graph, respectively), based on all possible orbits depicted in Figure 13A. The cost of aligning two nodes takes into consideration both their degrees and graphlet-signature similarity, whereas the lower cost is assigned to high degree nodes, as well as to graphlet similarities which do not replicate lower-degree graphlets. The starting seed node-pair with the lowest cost is chosen and the alignment is expanded outwards from these two nodes. In this example, nodes *V2*_*G*_ and *V1*_*A*_ have the highest degrees and the most similar graphlet-signatures, thus the pair (*V2*_*G*_*, V1*_*A*_) is chosen as the first aligned seed. The next highest degree node connected to *V2*_*G*_ is node *V2*_*I*_. Node *V2*_*I*_ is then randomly matched to one of the nodes *V1*_*B*_*, V1*_*C*_*, V1*_*D*_*, V1*_*E*_ (same degrees and similarity distances). For demonstration purposes let it be *V1*_*B*_. Continuing on this graph's path, node *V2*_*J*_ is matched to *V1*_*C*_. Moving on, node *V2*_*H*_ is aligned to node *V1*_*F*_ and finally node *V2*_*K*_ is randomly aligned to either *V1*_*D*_ or *V1*_*E*_. Let it be *V1*_*E*_. *V1*_*D*_ remains unaligned. The final GRAAL alignment is shown in Figure 13D.

## Link Prediction

Besides network alignment, predicting link changes in a single network has recently drawn attention in the biomedical field. Link prediction might concern the creation of future edges or the identification of missing links (e.g., incomplete data). While link prediction techniques are widely used by social media, in biological networks, they have also been used to identify potential drug side effects, protein-protein interactions, disease phenotypes based on molecular information and phylogenetic relations. For example, its application on bipartite graphs has unraveled new drug-target interactions (Kunegis et al., 2013). Its application on heterogeneous biological networks, has led to the identification of key pathway and protein interactions responsible for disease pathogenesis as well as candidate multiple sclerosis-associated genes (Himmelstein and Baranzini, 2015). Its combination with multi-way-based spectral has led to link prediction of protein–protein interaction networks.

The algorithm chosen for link prediction is often tied to the data type of the network. Due to the fact that each network type comes with its own growth pattern, relative assumptions must be made (Kunegis et al., 2013). Starting from an adjacency matrix *A*, through eigenvalue decomposition we can write *A* as *U*Λ*U*^*T*^. *U* is an *n* × *n* orthogonal matrix and Λ an *n* × *n* diagonal matrix. The values Λ*ii* are the eigenvalues of *A*, and the columns of *U* are its eigenvectors. The spectral evolution model states that in dynamic networks, eigenvalues change over time while eigenvectors remain constant. Some of the most common link prediction algorithm categories are listed below. *Triangle closing* or *triadic closure* (Leskovec et al., 2008) is a method for predicting edges which will appear between nodes with common neighbors and widely used in social network analysis. *Path counting* (Lü et al., 2009) is the extension of triangle closing, giving two nodes with further level neighbors an additional, lower link prediction score. *Graph kernels* (Smola and Kondor, 2003; Ito et al., 2005) are functions which describe the similarity between two nodes and are often used for link prediction.

**Figure 14:**
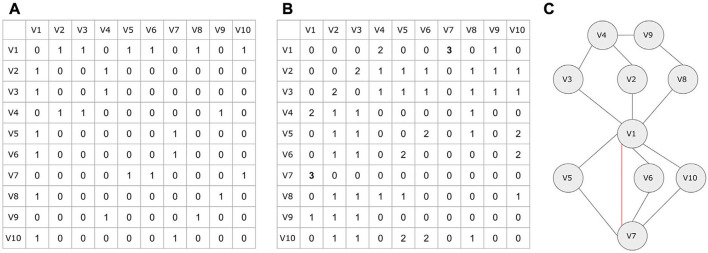
A triangle closing link prediction example. **(A)** The adjacency matrix of the undirected, unweighted example network. **(B)** The algebraic representation of the *A*^2^ matrix. Each (*i,j*) value represents the number of common neighbors of the nodes *i* and *j*. **(C)** The example network plot. The red edge represents the new predicted link in time point *t* + *1*.

Here, we demonstrate a link prediction example based on the triangle closing model. The adjacency matrix *A* of an unweighted and undirected network is shown in Figure 14A. The algebraic representation of the triangle closing model can be expressed as a square *A*^2^ of the adjacency matrix, where each (*i,j*) pair contains the number of common neighbors between *i* and *j* at a given time *t*. Thus, $A_{ij}^{2}=\underset{k}{\sum }A_{ik}A_{jk}.\;$The *A*^2^ matrix is shown in Figure 14B. Let the algorithm allow only one new edge creation at each iteration. At the time point *t*+*1*, the highest value of *A*^2^ is *3* (between nodes *V1* and *V7*). The new edge *V1–V7* is created and the *A*^2^ table is updated accordingly for the next iteration. If this edge already existed, the next highest value of *A*^2^ would be checked. In case of duplicate highest values, the algorithm would have chosen one of the corresponding edges to create randomly. The network of this example and the new edge created at time point *t*+*1* are depicted in Figure 14C.

Here, we present some tools which allow the computational link prediction on biological networks. HETIONET (Himmelstein and Baranzini, 2015) is an integrative biomedical knowledge network assembled from 29 different databases of genes, compounds, diseases, and more. Through HETIONET's website (https://het.io/), researchers can browse an interactive biological network of 47,031 nodes (11 types) and 2,250,197 relationships (24 types) and formulate their own edge predictions. Linkpred (Guns, 2014) calculates the likelihood of potential edge-creation in a future snapshot of a network. There are 18 predictor functions (local and global) to choose from. LPmade is another link prediction software which specializes in link prediction via commonly used unsupervised link prediction methods such as Adamic/Adar, common neighbors, Jaccard's coefficient, Katz, preferential attachment, PropFlow, rooted PageRank, SimRank, and weighted rooted PageRank.

## Network Perturbation

In biology, direct comparisons between a disease and a healthy state are very common, thus making the study of molecular changes essential. Therefore, at a network level, changes between such states are considered as biological network perturbations. In network medicine, a network's topology can be used as the backbone to further predict side effects in a system even at a 65–80% success rate (Santolini and Barabási, 2018). In the same study for example, a topology-based methodology was applied on a chemotaxis network of bacteria in order to predict the dynamics of perturbations such as gene knockout and overexpression with 90% accuracy. Furthermore, gene editing techniques such as CRISPR/Cas9 also benefit from network perturbation studies as the combination of single-cell sequencing methods with CRISPR/Cas9 offers detailed information of gene-knockout effects at a cellular level (Holding et al., 2019). Similar to gene knockout, RNA interference (RNAi) is a protein silencing method, where RNA molecules inhibit gene expression by targeting their mRNA. Nested effect models (NEMs) constitute probabilistic graphical models that describe the directed hierarchical dependencies on a perturbation network. In a recent study (Siebourg-Polster et al., 2015), it has been shown that an extended version of NEMs (NEMix), was proposed and used on signaling pathways' networks. In a use case scenario of a human rhinovirus (HRV) infection signaling network constructed from RNAi screening data, the proposed method inferred highly accurate signaling networks, fully aligned to the ones in KEGG database.

## Tools

Nowadays, a great variety of tools for network storage, analysis, and interactive visualization is available. Recent review articles (Pavlopoulos et al., 2008a, 2013, 2015, 2017; Gehlenborg et al., 2010; O'Donoghue et al., 2010) discuss the main challenges in the field in terms of storage and scalability and highlight the advantages and shortcomings of the current state-of-the-art tools. Briefly, Cytoscape (Shannon et al., 2003), Cytoscape.js (Franz et al., 2016), Gephi (Bastian et al., 2009), Pajek (Mrvar and Batagelj, 2016), Ondex (Köhler et al., 2006), Proviz (Iragne et al., 2005), VisANT (Hu et al., 2009), Medusa (Pavlopoulos et al., 2011b), Osprey (Breitkreutz et al., 2002), Arena3D (Pavlopoulos et al., 2008b; Secrier et al., 2012), Graphia (Kajeka), and BioLayout Express (Theocharidis et al., 2009) are a state-of-the-art of the tools worth mentioning. While many of them are designed for general use, most of them can be used to tackle problems in the network biology field. Ondex for example can integrate heterogeneous data from various sources, Gephi, Pajek, and Graphia (https://kajeka.com/graphia/) are interactive visualizers suitable for large-scale networks, Cytoscape hosts a great variety (>200) of plugins (Saito et al., 2012) and focuses on network visualization and annotation, Arena3D visualizes stacks of 2D networks in 3D space using a multi-layer concept and BioLayout Express and Graphia (Kajeka) are designed for 3D visualizations. Most of these tools (e.g., Gephi, Cytoscape, and Graphia) are highly interactive and allow network editing (node/edge coloring, size changing, labeling, annotations, zooming/rotating, collapse/expand grouping, arrow types, node/edge filtering, etc). In addition, tools such as the Network Analyzer (Doncheva et al., 2012), ZoomOut (Athanasiadis et al., 2015), Network Analysis Toolkit (NEAT) (Brohée et al., 2008), and NAP (Theodosiou et al., 2017) focus on the topological analysis, whereas non-interactive libraries such as the Stanford Network Analysis Project (SNAP) (Leskovec and Sosič, 2016), the outdated Large Graph Layout (LGL) (Adai et al., 2004), NetworkX (Hagberg et al., 2008), and GraphViz are command line applications able to offer back-end calculations as well as static visualizations. Specialized tools such as the Pathview (Luo and Brouwer, 2013), BioTapestry (Longabaugh, 2012), PathVisio (Kutmon et al., 2015), Interactive Pathways Explorer (iPath) (Darzi et al., 2018), MapMan (Thimm et al., 2004), and WikiPathways (Slenter et al., 2018), Pathway Commons (Rodchenkov et al., 2019) are designed for pathway analysis and visualization and finally, tools such as the Dendroscope (Huson et al., 2007) and iTOL (Letunic and Bork, 2007) are widely-used tree visualizers (Pavlopoulos et al., 2010). GeneMANIA (Franz et al., 2018) is offered for the detection of genes that are related to a set of input genes, using a very large set of functional association data (protein and genetic interactions, pathways, co-expression, co-localization, and protein domain similarity).

Concerning libraries, *igraph* (Gabor and Nepusz, 2006) is an open source library for network analysis and can be used by both Python and R languages. It offers a very rich plethora of functions dedicated to network analysis while it emphasizes on efficiency, portability, and ease of use. *VisNetwork* is a JavaScript-based R package for network visualization and *ggplot2* an R data visualization package suitable for interactive charts and plots. *Graphviz* is an open source graph visualization software for representing structural information such as diagrams of abstract graphs and networks. *NetworkD3* is a D3 JavaScript library and *plotly* a library suitable for data analytics. In addition, *Three.js* is a cross-browser JavaScript library for animated 3D computer graphics in a web browser and *ndtv-d3* a library suitable for timelines and animated movies of objects. *NetworkX* is a Python package for the creation, manipulation, and study of the structure, dynamics, and functions of complex networks and *Graph-tool* (Peixoto, 2017) a Python module for manipulation and statistical analysis of graphs. Finally, as biological network analyses become more and more popular, data exchange is crucial. For this purpose, the NDEx Project (Pillich et al., 2017) provides an open-source framework where scientists and organizations can share, store, manipulate, and publish biological network knowledge.

## Discussion

The adoption of mature high-throughput -Omics approaches to analyze biological samples (e.g., genomics, transcriptomics, proteomics, metabolomics etc.) has led to the production of data at the scale of tera- to peta-byte in size. Subsequently, due to this trend, biological networks follow an exponential growth, thus making their exploration, visualization, analysis, and storage a very difficult task. Therefore, traditional algorithms and data structures often fail to address scalability issues, thus making the adoption of modern technologies a necessity. As current tools are often limited in coping with large-scale datasets, Big Data approaches as well as parallel processing could be used for storing, querying, and processing large data volumes. Ideally, network analysis and visualization software could support algorithms which can run on distributed memory or multiple CPU and GPU systems for increased performance. In addition, global state-of-the-art data structures adjusted to such systems would be of great benefit.

Another bottleneck in systems biology is the visualization and representation of large-scale networks. As networks increase in size and complexity, more efficient algorithms for visualization are necessary. Notably, an alternative way to overcome 2D/3D space limitations is the adoption of virtual reality (VR) technologies. This way, biological networks could be for example explored or browsed using virtual universes. Typical examples for visualizing living systems such as a whole cell using such technology are the Visible Cell (Gagescu, 2001) or CELLmicrocosmos (Sommer, 2019). However, even after a decade of its existence, graphical limits, and cost of VR devices are still restrictive factors to be considered.

## Author Contributions

MK and EK wrote most of the manuscript. DP-E helped with the structure and with the biological content. GP conceived the concept and supervised the whole process.

### Conflict of Interest

The authors declare that the research was conducted in the absence of any commercial or financial relationships that could be construed as a potential conflict of interest.

## References

[B1] Adai A. T. Date S. V. Wieland S. Marcotte E. M. (2004). LGL: creating a map of protein function with an algorithm for visualizing very large biological networks. J. Mol. Biol. 340, 179–190. 10.1016/j.jmb.2004.04.047 15184029

[B2] Al-Anzi B. Arpp P. Gerges S. Ormerod C. Olsman N. Zinn K. (2015). Experimental and computational analysis of a large protein network that controls fat storage reveals the design principles of a signaling network. PLoS Comput. Biol. 11:e1004264. 10.1371/journal.pcbi.1004264 26020510 PMC4447291

[B3] Alexander S. Joydeep G. (2003). Cluster ensembles—a knowledge reuse framework for combining multiple partitions. J. Mach. Learn. Res. 3, 583–617. 10.1162/153244303321897735

[B4] Altschul S. F. Gish W. Miller W. Myers E. W. Lipman D. J. (1990). Basic local alignment search tool. J. Mol. Biol. 215, 403–410. 10.1016/S0022-2836(05)80360-2 2231712

[B5] Ana L. N. F. Jain A. K. (2003). Robust data clustering, in: IEEE Computer Society Conference on Computer Vision and Pattern Recognition, 2003 Proceedings (Madison, WI: IEEE Comput. Soc), II-128-II−133. Available online at: http://ieeexplore.ieee.org/document/1211462/ (accessed August 21, 2019).

[B6] Athanasiadis E. I. Bourdakou M. M. Spyrou G. M. (2015). Zoomout: analyzing multiple networks as single nodes. IEEE/ACM Trans. Comput. Biol. Bioinform. 12, 1213–1216. 10.1109/TCBB.2015.2424411 26451833

[B7] Auber D. Archambault D. Bourqui R. Delest M. Dubois J. Lambert A.. (2017). Tulip 5, in Encyclopedia of Social Network Analysis and Mining, eds Alhajj R. Rokne J. (New York, NY: Springer New York, 1–28.

[B8] Azad A. Pavlopoulos G. A. Ouzounis C. A. Kyrpides N. C. Buluç A. (2018). HipMCL: a high-performance parallel implementation of the Markov clustering algorithm for large-scale networks. Nucleic Acids Res. 46:e33. 10.1093/nar/gkx1313 29315405 PMC5888241

[B9] Bader G. D. Betel D. Hogue C. W. (2003). BIND: the biomolecular interaction network database. Nucleic Acids Res. 31, 248–250. 10.1093/nar/gkg056 12519993 PMC165503

[B10] Bader G. D. Hogue C. W. (2003). An automated method for finding molecular complexes in large protein interaction networks. BMC Bioinform. 4:2. 10.1186/1471-2105-4-2 12525261 PMC149346

[B11] Barabasi A.-L. Albert R. (1999). Emergence of scaling in random networks. Science 286, 509–512. 10.1126/science.286.5439.509 10521342

[B12] Barabási A.-L. Oltvai Z. N. (2004). Network biology: understanding the cell's functional organization. Nat. Rev. Genet. 5, 101–113. 10.1038/nrg1272 14735121

[B13] Barrett T. Wilhite S. E. Ledoux P. Evangelista C. Kim I. F. Tomashevsky M.. (2013). NCBI GEO: archive for functional genomics data sets–update. Nucleic Acids Res. 41, D991–D995. 10.1093/nar/gks1193 23193258 PMC3531084

[B14] Bastian M. Heymann S. Jacomy M. (2009). Gephi: an open source software for exploring and manipulating networks, in International AAAI Conference on Weblogs and Social Media. Available online at: http://www.aaai.org/ocs/index.php/ICWSM/09/paper/view/154

[B15] Baum B. R. (1989). PHYLIP: Phylogeny inference package. version 3.2. joel felsenstein. Q. Rev. Biol. 64, 539–41. 10.1086/416571

[B16] Bindea G. Mlecnik B. Hackl H. Charoentong P. Tosolini M. Kirilovsky A.. (2009). ClueGO: a cytoscape plug-in to decipher functionally grouped gene ontology and pathway annotation networks. Bioinformatics 25, 1091–1093. 10.1093/bioinformatics/btp101 19237447 PMC2666812

[B17] Blondel V. D. Guillaume J.-L. Lambiotte R. Lefebvre E. (2008). Fast unfolding of communities in large networks. J. Stat. Mech. Theory Exp. 2008:P10008. 10.1088/1742-5468/2008/10/P10008

[B18] Bollobás B. (2001). Random Graphs, 2nd Edn. Cambridge, NY: Cambridge University Press, 498. 10.1017/CBO9780511814068

[B19] Bonacich P. (1987). Power and centrality: a family of measures. Am. J. Sociol. 92, 1170–1182. 10.1086/228631

[B20] Bonnici V. Caro G. D. Constantino G. Liuni S. D'Elia D. Bombieri N.. (2018). Arena-Idb: a platform to build human non-coding RNA interaction networks. BMC Bioinform. 19 (Suppl. 10):350. 10.1186/s12859-018-2298-8 30367585 PMC6191940

[B21] Brandes U. Eiglsperger M. Lerner J. Pich C. (2017). Graph Markup Language (GraphML). Boca Raton, FL: Taylor & Francis, CRC Press, 517–541.

[B22] Breitkreutz B.-J. Stark C. Tyers M. (2002). Osprey: a network visualization system. Genome Biol. 3:PREPRINT0012. 10.1186/gb-2002-3-12-preprint0012 12537552

[B23] Brohée S. Faust K. Lima-Mendez G. Sand O. Janky R. Vanderstocken G.. (2008). NeAT: a toolbox for the analysis of biological networks, clusters, classes and pathways. Nucleic Acids Res. 36, W444–W451. 10.1093/nar/gkn336 18524799 PMC2447721

[B24] Brohée S. van Helden J. (2006). Evaluation of clustering algorithms for protein-protein interaction networks. BMC Bioinform. 7:488. 10.1186/1471-2105-7-488 17087821 PMC1637120

[B25] Chaouiya C. (2007). Petri net modelling of biological networks. Brief Bioinform. 8, 210–219. 10.1093/bib/bbm029 17626066

[B26] Chatr-aryamontri A. Ceol A. Palazzi L. M. Nardelli G. Schneider M. V. Castagnoli L.. (2007). MINT: the molecular INTeraction database. Nucleic Acids Res. 35, D572–D574. 10.1093/nar/gkl950 17135203 PMC1751541

[B27] Conant G. C. Wagner A. (2003). Convergent evolution of gene circuits. Nat. Genet. 34, 264–266. 10.1038/ng1181 12819781

[B28] Croft D. P. Krause J. James R. (2004). Social networks in the guppy (poecilia reticulata). Proc. Biol. Sci. 271 (Suppl. 6):S516–S519. 10.1098/rsbl.2004.0206 15801620 PMC1810091

[B29] Dagum L. Menon R. (1998). Open MP: an industry standard API for shared-memory programming. IEEE Comput. Sci. Eng. 5, 46–55. 10.1109/99.660313

[B30] Danon L. Ford A. P. House T. Jewell C. P. Keeling M. J. Roberts G. O.. (2011). Networks and the epidemiology of infectious disease. Interdiscip. Perspect Infect. Dis. 2011, 1–28. 10.1155/2011/284909 21437001 PMC3062985

[B31] Darzi Y. Letunic I. Bork P. Yamada T. (2018). iPath3.0: interactive pathways explorer v3. Nucleic Acids Res. 46, W510–W513. 10.1093/nar/gky299 29718427 PMC6031023

[B32] Delmas E. Besson M. Brice M.-H. Burkle L. A. Dalla Riva G. V. Fortin M.-J.. (2019). Analysing ecological networks of species interactions: analyzing ecological networks. Biol. Rev. 94, 16–36. 10.1111/brv.12433 29923657

[B33] Demir E. Cary M. P. Paley S. Fukuda K. Lemer C. Vastrik I.. (2010). The BioPAX community standard for pathway data sharing. Nat. Biotechnol. 28, 935–942. 10.1038/nbt.1666 20829833 PMC3001121

[B34] Doncheva N. T. Assenov Y. Domingues F. S. Albrecht M. (2012). Topological analysis and interactive visualization of biological networks and protein structures. Nat. Protoc. 7, 670–685. 10.1038/nprot.2012.004 22422314

[B35] Dongen S. (2000). Performance Criteria for Graph Clustering and Markov Cluster Experiments. National research institute for mathematics and computer science.

[B36] Eden E. Navon R. Steinfeld I. Lipson D. Yakhini Z. (2009). GOrilla: a tool for discovery and visualization of enriched GO terms in ranked gene lists. BMC Bioinform. 10:48. 10.1186/1471-2105-10-48 19192299 PMC2644678

[B37] Ekre A. R. Mante R. V. (2016). Genome sequence alignment tools: a review, in 2016 2nd International Conference on Advances in Electrical, Electronics, Information, Communication and Bio-Informatics (AEEICB) (Chennai: IEEE), 677–681. Available online at: http://ieeexplore.ieee.org/lpdocs/epic03/wrapper.htm?arnumber=7538378 (accessed July 18, 2019).

[B38] Enright A. J. Van Dongen S. Ouzounis C. A. (2002). An efficient algorithm for large-scale detection of protein families. Nucleic Acids Res. 30, 1575–1584. 10.1093/nar/30.7.1575 11917018 PMC101833

[B39] Fabregat A. Jupe S. Matthews L. Sidiropoulos K. Gillespie M. Garapati P.. (2018). The reactome pathway knowledgebase. Nucleic Acids Res. 46, D649–D655. 10.1093/nar/gkx1132 29145629 PMC5753187

[B40] Fagny M. Paulson J. N. Kuijjer M. L. Sonawane A. R. Chen C.-Y. Lopes-Ramos C. M.. (2017). Exploring regulation in tissues with eQTL networks. Proc. Natl. Acad. Sci. U.S.A. 114, E7841–E7850. 10.1073/pnas.1707375114 28851834 PMC5604022

[B41] Ferro A. Giugno R. Pigola G. Pulvirenti A. Skripin D. Bader G. D.. (2007). NetMatch: a cytoscape plugin for searching biological networks. Bioinform. Oxf. Engl. 23, 910–912. 10.1093/bioinformatics/btm032 17277332

[B42] Flannick J. Novak A. Do C. B. Srinivasan B. S. Batzoglou S. (2008). Automatic parameter learning for multiple network alignment, in Research in Computational Molecular Biology, eds Vingron M. Wong L. (Berlin: Springer Berlin Heidelberg), 214–231. Available online at: http://link.springer.com/10.1007/978-3-540-78839-3_19 (accessed December 16, 2019).

[B43] Forum M. P. I. (1994). MPI: A Message-Passing Interface. Oregon Graduate Institute School of Science & Engineering. Report No.: 890839.

[B44] Fowlkes E. B. Mallows C. L. (1983). A method for comparing two hierarchical clusterings. J. Am. Stat. Assoc. 78, 553–569. 10.1080/01621459.1983.10478008

[B45] Franceschini A. Szklarczyk D. Frankild S. Kuhn M. Simonovic M. Roth A.. (2013). STRING v9.1: protein-protein interaction networks, with increased coverage and integration. Nucleic Acids Res. 41, D808–D815. 10.1093/nar/gks1094 23203871 PMC3531103

[B46] Franz M. Lopes C. T. Huck G. Dong Y. Sumer O. Bader G. D. (2016). Cytoscape.js: a graph theory library for visualisation and analysis. Bioinform. Oxf. Engl. 32, 309–311. 10.1093/bioinformatics/btv557 26415722 PMC4708103

[B47] Franz M. Rodriguez H. Lopes C. Zuberi K. Montojo J. Bader G. D.. (2018). GeneMANIA update 2018. Nucleic Acids Res. 46, W60–W64. 10.1093/nar/gky311 29912392 PMC6030815

[B48] Freeman L. C. (1977). A set of measures of centrality based on betweenness. Sociometry 40, 35–41. 10.2307/3033543

[B49] Frey B. J. Dueck D. (2007). Clustering by passing messages between data points. Science 315, 972–976. 10.1126/science.1136800 17218491

[B50] Fruchterman T. M. J. Reingold E. M. (1991). Graph drawing by force-directed placement. Softw. Pract. Exp. 21, 1129–1164. 10.1002/spe.4380211102

[B51] Fung B. C. M. Wang K. Ester M. (2003). Hierarchical document clustering using frequent itemsets, in Proceedings of the 2003 SIAM International Conference on Data Mining (San Francisco, CA: Society for Industrial and Applied Mathematics), 59–70. Available online at: https://epubs.siam.org/doi/10.1137/1.9781611972733.6 (accessed September 2, 2019).

[B52] Gabor C. Nepusz T. (2006). The Igraph Software Package for Complex Network Research. InterJournal;Complex Systems:1695.

[B53] Gagescu R. (2001). The visible cell project. Nat. Rev. Mol. Cell Biol. 2, 231–231. 10.1038/35067039

[B54] Gagneur J. Jackson D. B. Casari G. (2003). Hierarchical analysis of dependency in metabolic networks. Bioinformatics 19, 1027–1034. 10.1093/bioinformatics/btg115 12761067

[B55] Gavin A.-C. Aloy P. Grandi P. Krause R. Boesche M. Marzioch M.. (2006). Proteome survey reveals modularity of the yeast cell machinery. Nature 440, 631–636. 10.1038/nature04532 16429126

[B56] Gehlenborg N. O'Donoghue S. I. Baliga N. S. Goesmann A. Hibbs M. A. Kitano H.. (2010). Visualization of omics data for systems biology. Nat. Methods 7, S56–S68. 10.1038/nmeth.1436 20195258

[B57] Gene Ontology Consortium (2004). The gene ontology (GO) database and informatics resource. Nucleic Acids Res. 32, 258D−261D. 10.1093/nar/gkh036 PMC308770 14681407

[B58] Gentleman R. C. Carey V. J. Bates D. M. Bolstad B. Dettling M. Dudoit S.. (2004). Bioconductor: open software development for computational biology and bioinformatics. Genome Biol. 5:R80. 10.1186/gb-2004-5-10-r80 15461798 PMC545600

[B59] Gioutlakis A. Klapa M. I. Moschonas N. K. (2017). PICKLE 2.0: a human protein-protein interaction meta-database employing data integration via genetic information ontology. PLoS ONE 12:e0186039. 10.1371/journal.pone.0186039 29023571 PMC5638325

[B60] Goh K.-I. Cusick M. E. Valle D. Childs B. Vidal M. Barabási A.-L. (2007). The human disease network. *Proc. Natl. Acad. Sci*. U.S.A. 104, 8685–8690. 10.1073/pnas.0701361104 PMC1885563 17502601

[B61] Golding B. Felsenstein J. (1990). A maximum likelihood approach to the detection of selection from a phylogeny. J. Mol. Evol. 31, 511–523. 10.1007/BF02102078 2176699

[B62] Gottlieb A. Stein G. Y. Ruppin E. Sharan R. (2011). PREDICT: a method for inferring novel drug indications with application to personalized medicine. Mol. Syst. Biol. 7:496. 10.1038/msb.2011.26 21654673 PMC3159979

[B63] Gu S. Johnson J. Faisal F. E. Milenković T. (2018). From homogeneous to heterogeneous network alignment via colored graphlets. Sci. Rep. 8:12524. 10.1038/s41598-018-30831-w 30131590 PMC6104050

[B64] Guns R. (2014). Link prediction, in Measuring Scholarly Impact, eds Ding Y. Rousseau R. Wolfram D. (Cham: Springer International Publishing), 35–55. Available online at: http://link.springer.com/10.1007/978-3-319-10377-8_2 (accessed December 17, 2019).

[B65] Hagberg A. Schult D. Swart P. (2008). Exploring network structure, dynamics, and function using network, in Proceedings of the 7th Python in Science Conference (Pasadena, CA: SciPy), 11–15.

[B66] Hage P. Harary F. (1995). Eccentricity and centrality in networks. Soc. Netw. 17, 57–63. 10.1016/0378-8733(94)00248-9

[B67] Han H. Cho J.-W. Lee S. Yun A. Kim H. Bae D.. (2018). TRRUST v2: an expanded reference database of human and mouse transcriptional regulatory interactions. Nucleic Acids Res. 46, D380–D386. 10.1093/nar/gkx1013 29087512 PMC5753191

[B68] Han H. Lee S. Lee I. (2019). NGSEA: network-based gene set enrichment analysis for interpreting gene expression phenotypes with functional gene sets. Mol. Cells 42, 579–588. 10.1101/636498 31307154 PMC6715341

[B69] Hermjakob H. Montecchi-Palazzi L. Bader G. Wojcik J. Salwinski L. Ceol A.. (2004b). The HUPO PSI's molecular interaction format—a community standard for the representation of protein interaction data. Nat. Biotechnol. 22, 177–183. 10.1038/nbt926 14755292

[B70] Hermjakob H. Montecchi-Palazzi L. Lewington C. Mudali S. Kerrien S. Orchard S.. (2004a). IntAct: an open source molecular interaction database. Nucleic Acids Res. 32, D452–D455. 10.1093/nar/gkh052 14681455 PMC308786

[B71] Himmelstein D. S. Baranzini S. E. (2015). Heterogeneous network edge prediction: a data integration approach to prioritize disease-associated genes. PLoS Comput. Biol. 11:e1004259. 10.1371/journal.pcbi.1004259 26158728 PMC4497619

[B72] Holding A. N. Cook H. V. Markowetz F. (2019). Data generation and network reconstruction strategies for single cell transcriptomic profiles of CRISPR-mediated gene perturbations. Biochim. Biophys. Acta BBA Gene Regul. Mech. 20:194441. 10.1016/j.bbagrm.2019.194441 31756390

[B73] Hu Z. Hung J.-H. Wang Y. Chang Y.-C. Huang C.-L. Huyck M.. (2009). VisANT 3.5: multi-scale network visualization, analysis and inference based on the gene ontology. Nucleic Acids Res. 37, W115–W121. 10.1093/nar/gkp406 19465394 PMC2703932

[B74] Huang D. W. Sherman B. T. Lempicki R. A. (2009a). Bioinformatics enrichment tools: paths toward the comprehensive functional analysis of large gene lists. Nucleic Acids Res. 37, 1–13. 10.1093/nar/gkn923 19033363 PMC2615629

[B75] Huang D. W. Sherman B. T. Lempicki R. A. (2009b). Systematic and integrative analysis of large gene lists using DAVID bioinformatics resources. Nat. Protoc. 4, 44–57. 10.1038/nprot.2008.211 19131956

[B76] Hucka M. Finney A. Sauro H. M. Bolouri H. Doyle J. C. Kitano H.. (2003). The systems biology markup language (SBML): a medium for representation and exchange of biochemical network models. Bioinform. Oxf. Engl. 19, 524–531. 10.1093/bioinformatics/btg015 12611808

[B77] Hung J.-H. Yang T.-H. Hu Z. Weng Z. DeLisi C. (2012). Gene set enrichment analysis: performance evaluation and usage guidelines. Brief Bioinform. 13, 281–291. 10.1093/bib/bbr049 21900207 PMC3357488

[B78] Huson D. H. Richter D. C. Rausch C. Dezulian T. Franz M. Rupp R. (2007). Dendroscope: an interactive viewer for large phylogenetic trees. BMC Bioinform. 8:460. 10.1186/1471-2105-8-460 18034891 PMC2216043

[B79] Huson D. H. Rupp R. Scornavacca C. (2010). Phylogenetic Networks: Concepts, Algorithms and Applications. Cambridge: Cambridge University Press. Available online at: http://ebooks.cambridge.org/ref/id/CBO9780511974076 (accessed July 22, 2019).

[B80] Ings T. C. Montoya J. M. Bascompte J. Blüthgen N. Brown L. Dormann C. F.. (2009). Ecological networks–beyond food webs. J. Anim. Ecol. 78, 253–269. 10.1111/j.1365-2656.2008.01460.x 19120606

[B81] Iragne F. Nikolski M. Mathieu B. Auber D. Sherman D. (2005). ProViz: protein interaction visualization and exploration. Bioinform. Oxf. Engl. 21, 272–274. 10.1093/bioinformatics/bth494 15347570

[B82] Ito T. Shimbo M. Kudo T. Matsumoto Y. (2005). Application of kernels to link analysis, in Proceeding of the Eleventh ACM SIGKDD International Conference on Knowledge Discovery in Data Mining - KDD'05 (Chicago, IL: ACM Press), 586. Available online at: http://portal.acm.org/citation.cfm?doid=1081870.1081941 (accessed December 17, 2019).

[B83] Jalili M. Salehzadeh-Yazdi A. Gupta S. Wolkenhauer O. Yaghmaie M. Resendis-Antonio O.. (2016). Evolution of centrality measurements for the detection of essential proteins in biological networks. Front. Physiol. 7:375. 10.3389/fphys.2016.00375 27616995 PMC4999434

[B84] Jeong H. Tombor B. Albert R. Oltvai Z. N. Barabási A. L. (2000). The large-scale organization of metabolic networks. Nature 407, 651–654. 10.1038/35036627 11034217

[B85] Jiang P. Singh M. (2010). SPICi: a fast clustering algorithm for large biological networks. Bioinform. Oxf. Engl. 26, 1105–1111. 10.1093/bioinformatics/btq078 20185405 PMC2853685

[B86] Junker B. H. Schreiber F. (eds) (2008). Analysis of Biological Networks, Wiley Series on Bioinformatics (Hoboken, NJ: Wiley-Interscience), 346. 10.1002/9780470253489

[B87] Kalaev M. Smoot M. Ideker T. Sharan R. (2008). NetworkBLAST: comparative analysis of protein networks. Bioinformatics 24, 594–596. 10.1093/bioinformatics/btm630 18174180

[B88] Kandasamy K. Mohan S. Raju R. Keerthikumar S. Kumar G. S. S. Venugopal A. K.. (2010). NetPath: a public resource of curated signal transduction pathways. Genome Biol. 11:R3. 10.1186/gb-2010-11-1-r3 20067622 PMC2847715

[B89] Kanehisa M. Goto S. (2000). KEGG: kyoto encyclopedia of genes and genomes. Nucleic Acids Res. 28, 27–30. 10.1093/nar/28.1.27 10592173 PMC102409

[B90] Kashtan N. Itzkovitz S. Milo R. Alon U. (2004). Efficient sampling algorithm for estimating subgraph concentrations and detecting network motifs. Bioinform. Oxf. Engl. 20, 1746–1758. 10.1093/bioinformatics/bth163 15001476

[B91] Kavurucu Y. (2015). A comparative study on network motif discovery algorithms. Int. J. Data Min. Bioinforma. 11, 180–204. 10.1504/IJDMB.2015.066777 26255382

[B92] Kiełbasa S. M. Wan R. Sato K. Horton P. Frith M. C. (2011). Adaptive seeds tame genomic sequence comparison. Genome Res. 21, 487–493. 10.1101/gr.113985.110 21209072 PMC3044862

[B93] Kim W. Li M. Wang J. Pan Y. (2011). Biological network motif detection and evaluation. BMC Syst. Biol. 5 (Suppl. 3):S5. 10.1186/1752-0509-5-S3-S5 22784624 PMC3287573

[B94] Kirch W. (ed). (2008). Pearson's Correlation Coefficient. In: Encyclopedia of Public Health (Dordrecht: Springer Netherlands), 1090–1. Available online at: http://link.springer.com/10.1007/978-1-4020-5614-7_2569 (accessed November 12, 2019).

[B95] Knuth D. E. (1997). The Art of Computer Programming, 3rd Edn. Reading: Addison-Wesle.

[B96] Köhler J. Baumbach J. Taubert J. Specht M. Skusa A. Rüegg A.. (2006). Graph-based analysis and visualization of experimental results with ONDEX. Bioinform. Oxf. Engl. 22, 1383–1390. 10.1093/bioinformatics/btl081 16533819

[B97] Koschützki D. Schreiber F. (2008). Centrality analysis methods for biological networks and their application to gene regulatory networks. Gene Regul. Syst. Biol. 2, 193–201. 10.4137/GRSB.S702 19787083 PMC2733090

[B98] Koyutürk M. Kim Y. Topkara U. Subramaniam S. Szpankowski W. Grama A. (2006). Pairwise alignment of protein interaction networks. J. Comput. Biol. 13, 182–199. 10.1089/cmb.2006.13.182 16597234

[B99] Kramer F. Bayerlová M. Klemm F. Bleckmann A. Beissbarth T. (2013). rBiopaxParser–an R package to parse, modify and visualize BioPAX data. Bioinform. Oxf. Engl. 29, 520–522. 10.1093/bioinformatics/bts710 23274212

[B100] Krzywinski M. Birol I. Jones S. J. Marra M. A. (2012). Hive plots–rational approach to visualizing networks. Brief Bioinform. 13, 627–644. 10.1093/bib/bbr069 22155641

[B101] Krzywinski M. Schein J. Birol I. Connors J. Gascoyne R. Horsman D.. (2009). Circos: an information aesthetic for comparative genomics. Genome Res. 19, 1639–1645. 10.1101/gr.092759.109 19541911 PMC2752132

[B102] Kuchaiev O. Milenković T. Memišević V. Hayes W. PrŽulj N. (2010). Topological network alignment uncovers biological function and phylogeny. J. R. Soc. Interface 7, 1341–1354. 10.1098/rsif.2010.0063 20236959 PMC2894889

[B103] Kumar S. Stecher G. Tamura K. (2016). MEGA7: molecular evolutionary genetics analysis version 7.0 for bigger datasets. Mol. Biol. Evol. 33, 1870–1874. 10.1093/molbev/msw054 27004904 PMC8210823

[B104] Kunegis J. Fay D. Bauckhage C. (2013). Spectral evolution in dynamic networks. Knowl. Inf. Syst. 37, 1–36. 10.1007/s10115-012-0575-9

[B105] Kutmon M. van Iersel M. P. Bohler A. Kelder T. Nunes N. Pico A. R.. (2015). PathVisio 3: an extendable pathway analysis toolbox. PLoS Comput. Biol. 11:e1004085. 10.1371/journal.pcbi.1004085 25706687 PMC4338111

[B106] Langfelder P. Zhang B. Horvath S. (2008). Defining clusters from a hierarchical cluster tree: the dynamic tree cut package for R. Bioinformatics 24, 719–720. 10.1093/bioinformatics/btm563 18024473

[B107] Le Novère N. Hucka M. Mi H. Moodie S. Schreiber F. Sorokin A.. (2009). Erratum: the systems biology graphical notation. Nat. Biotechnol. 27, 864–864. 10.1038/nbt0909-864d 19668183

[B108] Lehne B. Schlitt T. (2009). Protein-protein interaction databases: keeping up with growing interactomes. Hum. Genom. 3, 291–297. 10.1186/1479-7364-3-3-291 19403463 PMC3500230

[B109] Leskovec J. Backstrom L. Kumar R. Tomkins A. (2008). Microscopic evolution of social networks, in Proceeding of the 14th ACM SIGKDD International Conference on Knowledge Discovery and Data Mining - KDD 08 (Las Vegas, NV: ACM Press), 462. Available online at: http://dl.acm.org/citation.cfm?doid=1401890.1401948 (accessed December 17, 2019).

[B110] Leskovec J. Sosič R. (2016). SNAP: a general-purpose network analysis and graph-mining library. ACM Trans. Intell. Syst. Technol. 8, 1–20. 10.1145/2898361 28344853 PMC5361061

[B111] Letunic I. Bork P. (2007). Interactive tree of life (iTOL): an online tool for phylogenetic tree display and annotation. Bioinformatics 23, 127–128. 10.1093/bioinformatics/btl529 17050570

[B112] Li T. Ogihara M. Ma S. (2004). On combining multiple clusterings, in: Proceedings of the Thirteenth ACM Conference on Information and Knowledge Management - CIKM'04. (Washington, DC: ACM Press), 294. Available online at: http://portal.acm.org/citation.cfm?doid=1031171.1031234 (accessed August 21, 2019).

[B113] Lloyd C. M. Halstead M. D. B. Nielsen P. F. (2004). CellML: its future, present and past. Prog. Biophys. Mol. Biol. 85, 433–450. 10.1016/j.pbiomolbio.2004.01.004 15142756

[B114] Longabaugh W. J. R. (2012). BioTapestry: a tool to visualize the dynamic properties of gene regulatory networks. Methods Mol. Biol. 786, 359–394. 10.1007/978-1-61779-292-2_21 21938637

[B115] Lü L. Jin C. H. Zhou T. (2009). Similarity index based on local paths for link prediction of complex networks. Phys. Rev. E 80:046122. 10.1103/PhysRevE.80.046122 19905405

[B116] Luo W. Brouwer C. (2013). Pathview: an R/Bioconductor package for pathway-based data integration and visualization. Bioinformatics 29, 1830–1831. 10.1093/bioinformatics/btt285 23740750 PMC3702256

[B117] Ma H.-W. Zeng A.-P. (2003). The connectivity structure, giant strong component and centrality of metabolic networks. Bioinform. Oxf. Engl. 19, 1423–1430. 10.1093/bioinformatics/btg177 12874056

[B118] Maere S. Heymans K. Kuiper M. (2005). BiNGO: a cytoscape plugin to assess overrepresentation of gene ontology categories in biological networks. Bioinform. Oxf. Engl. 21, 3448–3449. 10.1093/bioinformatics/bti551 15972284

[B119] Mamano N. Hayes W. B. (2017). SANA: simulated annealing far outperforms many other search algorithms for biological network alignment. Bioinform. Oxf. Engl. 33, 2156–2164. 10.1093/bioinformatics/btx090 28203713

[B120] Mangan S. Alon U. (2003). Structure and function of the feed-forward loop network motif. Proc. Natl. Acad. Sci. U.S.A. 100, 11980–11985. 10.1073/pnas.2133841100 14530388 PMC218699

[B121] Mangan S. Zaslaver A. Alon U. (2003). The coherent feedforward loop serves as a sign-sensitive delay element in transcription networks. J. Mol. Biol. 334, 197–204. 10.1016/j.jmb.2003.09.049 14607112

[B122] Marina Meil ă. David H. (2001). An experimental comparison of model-based clustering methods. Mach. Learn. 42, 9–29. 10.1023/A:1007648401407

[B123] Martin S. Brown W. M. Klavans R. Boyack K. W. (2011). OpenOrd: An Open-Source Toolbox for Large Graph Layout. San Francisco: CA. Available from: http://proceedings.spiedigitallibrary.org/proceeding.aspx?doi=10.1117/12.871402 (accessed October 25, 2018).

[B124] Matys V. Fricke E. Geffers R. Gössling E. Haubrock M. Hehl R.. (2003). TRANSFAC: transcriptional regulation, from patterns to profiles. Nucleic Acids Res. 31, 374–378. 10.1093/nar/gkg108 12520026 PMC165555

[B125] McGillivray P. Clarke D. Meyerson W. Zhang J. Lee D. Gu M.. (2018). Network analysis as a grand unifier in biomedical data science. Annu. Rev. Biomed. Data Sci. 1, 153–180. 10.1146/annurev-biodatasci-080917-013444

[B126] Meila M. (2000). Comparing Clustering. University of Washington.

[B127] Merico D. Isserlin R. Stueker O. Emili A. Bader G. D. (2010). Enrichment map: a network-based method for gene-set enrichment visualization and interpretation. PLoS ONE 5:e13984. 10.1371/journal.pone.0013984 21085593 PMC2981572

[B128] Mi H. Muruganujan A. Casagrande J. T. Thomas P. D. (2013). Large-scale gene function analysis with the PANTHER classification system. Nat. Protoc. 8, 1551–1566. 10.1038/nprot.2013.092 23868073 PMC6519453

[B129] Michael L. S. A. (2017). DESeq2. Bioconductor. Available online at: https://bioconductor.org/packages/DESeq2 (accessed December 21, 2019).

[B130] Milenković T. Ng W. L. Hayes W. PrŽUlj N. (2010). Optimal network alignment with graphlet degree vectors. Cancer Inform. 9:S4744. 10.4137/CIN.S4744 20628593 PMC2901631

[B131] Milner-Gulland E. J. (2012). Interactions between human behaviour and ecological systems. Philos. Trans. R. Soc. Lond. B Biol. Sci. 367, 270–278. 10.1098/rstb.2011.0175 22144389 PMC3223800

[B132] Milo R. (2002). Network motifs: simple building blocks of complex networks. Science 298, 824–827. 10.1126/science.298.5594.824 12399590

[B133] Mirkin B. (2001). Eleven ways to look at the chi-squared coefficient for contingency tables. Am. Stat. 55, 111–120. 10.1198/000313001750358428

[B134] Mootha V. K. Lindgren C. M. Eriksson K.-F. Subramanian A. Sihag S. Lehar J.. (2003). PGC-1alpha-responsive genes involved in oxidative phosphorylation are coordinately downregulated in human diabetes. Nat. Genet. 34, 267–273. 10.1038/ng1180 12808457

[B135] Moreau Y. Tranchevent L.-C. (2012). Computational tools for prioritizing candidate genes: boosting disease gene discovery. Nat. Rev. Genet. 13, 523–536. 10.1038/nrg3253 22751426

[B136] Morris J. H. Apeltsin L. Newman A. M. Baumbach J. Wittkop T. Su G.. (2011). Clustermaker: a multi-algorithm clustering plugin for cytoscape. BMC Bioinform. 12:436. 10.1186/1471-2105-12-436 22070249 PMC3262844

[B137] Moschopoulos C. N. Pavlopoulos G. A. Iacucci E. Aerts J. Likothanassis S. Schneider R.. (2011). Which clustering algorithm is better for predicting protein complexes? BMC Res. Notes 4:549. 10.1186/1756-0500-4-549 22185599 PMC3267700

[B138] Moulos P. Hatzis P. (2015). Systematic integration of RNA-Seq statistical algorithms for accurate detection of differential gene expression patterns. Nucleic Acids Res. 43:e25. 10.1093/nar/gku1273 25452340 PMC4344485

[B139] Mrvar A. Batagelj V. (2016). Analysis and visualization of large networks with program package Pajek. Comp. Adapt. Syst. Model 4:6. 10.1186/s40294-016-0017-8

[B140] Mudunuri U. Che A. Yi M. Stephens R. M. (2009). bioDBnet: the biological database network. Bioinform. Oxf. Engl. 25, 555–556. 10.1093/bioinformatics/btn654 19129209 PMC2642638

[B141] Murray-Rust P. Rzepa H. S. Wright M. (2001). Development of chemical markup language (CML) as a system for handling complex chemical content. N. J. Chem. 25, 618–634. 10.1039/b008780g

[B142] Navlakha S. Kingsford C. (2010). The power of protein interaction networks for associating genes with diseases. Bioinform. Oxf. Engl. 26, 1057–1063. 10.1093/bioinformatics/btq076 20185403 PMC2853684

[B143] Needleman S. B. Wunsch C. D. (1970). A general method applicable to the search for similarities in the amino acid sequence of two proteins. J. Mol. Biol. 48, 443–453. 10.1016/0022-2836(70)90057-4 5420325

[B144] Newman M. E. J. Girvan M. (2004). Finding and evaluating community structure in networks. Phys. Rev. E 69:026113. 10.1103/PhysRevE.69.026113 14995526

[B145] Ni J. Koyuturk M. Tong H. Haines J. Xu R. Zhang X. (2016). Disease gene prioritization by integrating tissue-specific molecular networks using a robust multi-network model. BMC Bioinform. 17:453. 10.1186/s12859-016-1317-x 27829360 PMC5103411

[B146] Nica A. C. Dermitzakis E. T. (2013). Expression quantitative trait loci: present and future. Philos. Trans. R. Soc. B Biol. Sci. 368:20120362. 10.1098/rstb.2012.0362 23650636 PMC3682727

[B147] O'Donoghue S. I. Gavin A.-C. Gehlenborg N. Goodsell D. S. Hériché J.-K Nielsen C. B.. (2010). Visualizing biological data-now and in the future. Nat. Methods 7, S2–S4. 10.1038/nmeth.f.301 20195254

[B148] Pafilis E. Buttigieg P. L. Ferrell B. Pereira E. Schnetzer J. Arvanitidis C.. (2016). EXTRACT: interactive extraction of environment metadata and term suggestion for metagenomic sample annotation. Database J. Biol. Databases Curat. 2016:baw005. 10.1093/database/baw005 26896844 PMC4761108

[B149] Parkinson H. Kapushesky M. Shojatalab M. Abeygunawardena N. Coulson R. Farne A.. (2007). ArrayExpress–a public database of microarray experiments and gene expression profiles. Nucleic Acids Res. 35, D747–D750. 10.1093/nar/gkl995 17132828 PMC1716725

[B150] Pavlopoulos G. A. Hooper S. D. Sifrim A. Schneider R. Aerts J. (2011b). Medusa: a tool for exploring and clustering biological networks. BMC Res. Notes 4:384. 10.1186/1756-0500-4-384 21978489 PMC3197509

[B151] Pavlopoulos G. A. Iacucci E. Iliopoulos I. Bagos P. (2013). Interpreting the omics ‘era' data, in Multimedia Services in Intelligent Environments, eds Tsihrintzis G. A. Virvou M. Jain L. C. (Heidelberg: Springer International Publishing), 79–100. Available online at: http://link.springer.com/10.1007/978-3-319-00375-7_6 (accessed January 13, 2019).

[B152] Pavlopoulos G. A. Kontou P. I. Pavlopoulou A. Bouyioukos C. Markou E. Bagos P. G. (2018). Bipartite graphs in systems biology and medicine: a survey of methods and applications. GigaScience 7, 1–31. 10.1093/gigascience/giy014 29648623 PMC6333914

[B153] Pavlopoulos G. A. Malliarakis D. Papanikolaou N. Theodosiou T. Enright A. J. Iliopoulos I. (2015). Visualizing genome and systems biology: technologies, tools, implementation techniques and trends, past, present and future. GigaScience 4:38. 10.1186/s13742-015-0077-2 26309733 PMC4548842

[B154] Pavlopoulos G. A. O'Donoghue S. I. Satagopam V. P. Soldatos T. G. Pafilis E. Schneider R. (2008b). Arena3D: visualization of biological networks in 3D. BMC Syst. Biol. 2:104. 10.1186/1752-0509-2-104 19040715 PMC2637860

[B155] Pavlopoulos G. A. Paez-Espino D. Kyrpides N. C. Iliopoulos I. (2017). Empirical comparison of visualization tools for larger-scale network analysis. Adv. Bioinforma. 2017:1278932. 10.1155/2017/1278932 28804499 PMC5540468

[B156] Pavlopoulos G. A. Promponas V. J. Ouzounis C. A. Iliopoulos I. (2014). Biological information extraction and co-occurrence analysis. Methods Mol. Biol. 1159, 77–92. 10.1007/978-1-4939-0709-0_5 24788262

[B157] Pavlopoulos G. A. Secrier M. Moschopoulos C. N. Soldatos T. G. Kossida S. Aerts J.. (2011a). Using graph theory to analyze biological networks. BioData Min. 4:10. 10.1186/1756-0381-4-10 21527005 PMC3101653

[B158] Pavlopoulos G. A. Soldatos T. G. Barbosa-Silva A. Schneider R. (2010). A reference guide for tree analysis and visualization. BioData Min. 3:1. 10.1186/1756-0381-3-1 20175922 PMC2844399

[B159] Pavlopoulos G. A. Wegener A.-L. Schneider R. (2008a). A survey of visualization tools for biological network analysis. BioData Min. 1:12. 10.1186/1756-0381-1-12 19040716 PMC2636684

[B160] Pearson W. R. (2000). Flexible sequence similarity searching with the FASTA3 program package. Methods Mol. Biol. 132, 185–219. 10.1385/1-59259-192-2:185 10547837

[B161] Peixoto T. P. (2017). The Graph-Tool Python Library. Figshare. Available from: https://figshare.com/articles/graph_tool/1164194 (accessed December 19, 2019).

[B162] Peri S. Navarro J. D. Amanchy R. Kristiansen T. Z. Jonnalagadda C. K. Surendranath V.. (2003). Development of human protein reference database as an initial platform for approaching systems biology in humans. Genome Res. 13, 2363–2371. 10.1101/gr.1680803 14525934 PMC403728

[B163] Pillich R. T. Chen J. Rynkov V. Welker D. Pratt D. (2017). NDEx: a community resource for sharing and publishing of biological networks. Methods Mol. Biol. 1558, 271–301. 10.1007/978-1-4939-6783-4_13 28150243

[B164] Platig J. Castaldi P. J. DeMeo D. Quackenbush J. (2016). Bipartite community structure of eQTLs. PLoS Comput Biol. 12:e1005033. 10.1371/journal.pcbi.1005033 27618581 PMC5019382

[B165] Przulj N. Corneil D. G. Jurisica I. (2004). Modeling interactome: scale-free or geometric? Bioinform. Oxf. Engl. 20, 3508–3515. 10.1093/bioinformatics/bth436 15284103

[B166] Rand W. M. (1971). Objective Criteria for the evaluation of clustering methods. J. Am. Stat. Assoc. 66, 846–850. 10.1080/01621459.1971.10482356

[B167] Rao V. S. Srinivas K. Sujini G. N. Kumar G. N. S. (2014)Protein-protein interaction detection: methods analysis. Int. J. Proteomics 2014:1–12. 10.1155/2014/147648 PMC3947875 24693427

[B168] Raudvere U. Kolberg L. Kuzmin I. Arak T. Adler P. Peterson H.. (2019). g:Profiler: a web server for functional enrichment analysis and conversions of gene lists (2019 update). Nucleic Acids Res. 47, W191–W198. 10.1093/nar/gkz369 31066453 PMC6602461

[B169] Reimand J. Isserlin R. Voisin V. Kucera M. Tannus-Lopes C. Rostamianfar A.. (2019). Pathway enrichment analysis and visualization of omics data using g:Profiler, GSEA, cytoscape and enrichmentmap. Nat. Protoc. 14, 482–517. 10.1038/s41596-018-0103-9 30664679 PMC6607905

[B170] Reisig W. (1985). Petri Nets: An Introduction. Berlin, NY: Springer-Verlag. 161 (EATCS monographs on theoretical computer science). 10.1007/978-3-642-69968-9

[B171] Rodchenkov I. Babur O. Luna A. Aksoy B. A. Wong J. V. Fong D.. (2019). Pathway commons 2019 update: integration, analysis and exploration of pathway data. Nucleic Acids Res. 48, D489–D497. 10.1093/nar/gkz946 31647099 PMC7145667

[B172] Romanuk T. N. Vogt R. J. Young A. Tuck C. Carscallen M. W. (2010). Maintenance of positive diversity-stability relations along a gradient of environmental stress. PLoS ONE 5:e10378. 10.1371/journal.pone.0010378 20436913 PMC2860506

[B173] Sabidussi G. (1966). The centrality of a graph. Psychometrika 31, 581–603. 10.1007/BF02289527 5232444

[B174] Saito R. Smoot M. E. Ono K. Ruscheinski J. Wang P.-L. Lotia S.. (2012). A travel guide to cytoscape plugins. Nat. Methods 9, 1069–1076. 10.1038/nmeth.2212 23132118 PMC3649846

[B175] Saitou N. Nei M. (1987). The neighbor-joining method: a new method for reconstructing phylogenetic trees. Mol. Biol. Evol. 4, 406–425. 3447015 10.1093/oxfordjournals.molbev.a040454

[B176] Santolini M. Barabási A.-L. (2018). Predicting perturbation patterns from the topology of biological networks. Proc. Natl. Acad. Sci. U.S.A. 115, E6375–E6383. 10.1073/pnas.1720589115 29925605 PMC6142275

[B177] Satagopam V. P. Theodoropoulou M. C. Stampolakis C. K. Pavlopoulos G. A. Papandreou N. C. Bagos P. G.. (2010). GPCRs, G-proteins, effectors and their interactions: human-gpDB, a database employing visualization tools and data integration techniques. Database J. Biol. Databases Curat. 2010:baq019. 10.1093/database/baq019 20689020 PMC2931634

[B178] Schreiber F. Schwöbbermeyer H. (2005). MAVisto: a tool for the exploration of network motifs. Bioinform. Oxf. Engl. 21, 3572–3574. 10.1093/bioinformatics/bti556 16020473

[B179] Secrier M. Pavlopoulos G. A. Aerts J. Schneider R. (2012). Arena3D: visualizing time-driven phenotypic differences in biological systems. BMC Bioinform. 13:45. 10.1186/1471-2105-13-45 22439608 PMC3368716

[B180] Shannon P. Markiel A. Ozier O. Baliga N. S. Wang J. T. Ramage D.. (2003). Cytoscape: a software environment for integrated models of biomolecular interaction networks. Genome Res. 13, 2498–2504. 10.1101/gr.1239303 14597658 PMC403769

[B181] Sharan R. Suthram S. Kelley R. M. Kuhn T. McCuine S. Uetz P.. (2005). Conserved patterns of protein interaction in multiple species. Proc. Natl. Acad. Sci. U.S.A. 102, 1974–1979. 10.1073/pnas.0409522102 15687504 PMC548573

[B182] Sharan R. Ulitsky I. Shamir R. (2007). Network-based prediction of protein function. Mol. Syst. Biol. 3:88. 10.1038/msb4100129 17353930 PMC1847944

[B183] Shen-Orr S. S. Milo R. Mangan S. Alon U. (2002). Network motifs in the transcriptional regulation network of *Escherichia coli*. Nat. Genet. 31, 64–68. 10.1038/ng881 11967538

[B184] Siebourg-Polster J. Mudrak D. Emmenlauer M. Rämö. P Dehio C. Greber U.. (2015). NEMix: single-cell nested effects models for probabilistic pathway stimulation. PLoS Comput. Biol. 11:e1004078. 10.1371/journal.pcbi.1004078 25879530 PMC4400057

[B185] Slenter D. N. Kutmon M. Hanspers K. Riutta A. Windsor J. Nunes N.. (2018). WikiPathways: a multifaceted pathway database bridging metabolomics to other omics research. Nucleic Acids Res. 46, D661–D667. 10.1093/nar/gkx1064 29136241 PMC5753270

[B186] Smith T. F. Waterman M. S. (1981). Identification of common molecular subsequences. J. Mol. Biol. 147, 195–197. 10.1016/0022-2836(81)90087-5 7265238

[B187] Smola A. J. Kondor R. (2003). Kernels and regularization on graphs, in Learning Theory and Kernel Machines, eds Schölkopf B. Warmuth M. K. (Berlin: Springer Berlin Heidelberg), 144–158. Available online at: http://link.springer.com/10.1007/978-3-540-45167-9_12 (accessed December 17, 2019).

[B188] Sommer B. (2019). The CELLmicrocosmos tools: a small history of java-based cell and membrane modelling open source software development. J. Integr. Bioinform. 16:20190057. 10.1515/jib-2019-0057 31560649 PMC6798854

[B189] Sonawane A. R. Weiss S. T. Glass K. Sharma A. (2019). Network medicine in the age of biomedical big data. *Front*. Genet. 10:294. 10.3389/fgene.2019.00294 PMC6470635 31031797

[B190] Spirin V. Mirny L. A. (2003). Protein complexes and functional modules in molecular networks. Proc. Natl. Acad. Sci. U.S.A. 100, 12123–12128. 10.1073/pnas.2032324100 14517352 PMC218723

[B191] Stark C. Breitkreutz B.-J. Reguly T. Boucher L. Breitkreutz A. Tyers M. (2006). BioGRID: a general repository for interaction datasets. Nucleic Acids Res. 34, D535–D539. 10.1093/nar/gkj109 16381927 PMC1347471

[B192] Stone L. Simberloff D. Artzy-Randrup Y. (2019). Network motifs and their origins. PLoS Comput. Biol. 15:e1006749. 10.1371/journal.pcbi.1006749 30973867 PMC6459481

[B193] Subramanian A. Tamayo P. Mootha V. K. Mukherjee S. Ebert B. L. Gillette M. A.. (2005). Gene set enrichment analysis: a knowledge-based approach for interpreting genome-wide expression profiles. Proc. Natl. Acad. Sci. U.S.A. 102, 15545–15550. 10.1073/pnas.0506580102 16199517 PMC1239896

[B194] Szklarczyk D. Jensen L. J. (2015). Protein-protein interaction databases, in Protein-Protein Interactions, eds Meyerkord C. L. Fu H. (New York, NY: Springer New York), 39–56. 10.1007/978-1-4939-2425-7_3 25859942

[B195] Szklarczyk D. Santos A. von Mering C. Jensen L. J. Bork P. Kuhn M. (2016). STITCH 5: augmenting protein-chemical interaction networks with tissue and affinity data. Nucleic Acids Res. 44, D380–D384. 10.1093/nar/gkv1277 26590256 PMC4702904

[B196] Theocharidis A. van Dongen S. Enright A. J. Freeman T. C. (2009). Network visualization and analysis of gene expression data using biolayout express(3D). Nat. Protoc. 4, 1535–1550. 10.1038/nprot.2009.177 19798086

[B197] Theodosiou T. Efstathiou G. Papanikolaou N. Kyrpides N. C. Bagos P. G. Iliopoulos I.. (2017). NAP: The network analysis profiler, a web tool for easier topological analysis and comparison of medium-scale biological networks. BMC Res. Notes 10:278. 10.1186/s13104-017-2607-8 28705239 PMC5513407

[B198] Thimm O. Bläsing O. Gibon Y. Nagel A. Meyer S. Krüger P.. (2004). MAPMAN: a user-driven tool to display genomics data sets onto diagrams of metabolic pathways and other biological processes. Plant J. Cell Mol. Biol. 37, 914–939. 10.1111/j.1365-313X.2004.02016.x 14996223

[B199] Thomas R. Portier C. J. (2013). Gene expression networks. Methods Mol. Biol. 930, 165–178. 10.1007/978-1-62703-059-5_7 23086841

[B200] Tian W. Samatova N. F. (2008). Pairwise alignment of interaction networks by fast identification of maximal conserved patterns, in Biocomputing 2009 (Kohala Coast, HI: World Scientific), 99–110. Available online at: http://www.worldscientific.com/doi/abs/10.1142/9789812836939_0010 (accessed December 16, 2019). 19209698

[B201] Tipney H. Hunter L. (2010). An introduction to effective use of enrichment analysis software. Hum. Genomics 4, 202–206. 10.1186/1479-7364-4-3-202 20368141 PMC3525973

[B202] Torres J. M. Gamazon E. R. Parra E. J. Below J. E. Valladares-Salgado A. Wacher N.. (2014). Cross-tissue and tissue-specific eQTLs: partitioning the heritability of a complex trait. Am. J. Hum. Genet. 95, 521–534. 10.1016/j.ajhg.2014.10.001 25439722 PMC4225593

[B203] Towfic F. Greenlee M. H. W. Honavar V. (2009). Aligning biomolecular networks using modular graph kernels, in Algorithms in Bioinformatics, eds Salzberg S. L. Warnow T. (Berlin: Springer Berlin Heidelberg), 345–361. Available online at: http://link.springer.com/10.1007/978-3-642-04241-6_29 (accessed December 11, 2019).

[B204] Tripathi K. P. Evangelista D. Zuccaro A. Guarracino M. R. (2015). Transcriptator: an automated computational pipeline to annotate assembled reads and identify non coding RNA. PLoS ONE 10:e0140268. 10.1371/journal.pone.0140268 26581084 PMC4651556

[B205] Ulgen E. Ozisik O. Sezerman O. U. (2019). pathfindR: An R package for comprehensive identification of enriched pathways in omics data through active subnetworks. Front. Genet. 10:858. 10.3389/fgene.2019.00858 31608109 PMC6773876

[B206] Ulrich L. E. Zhulin I. B. (2007). MiST: a microbial signal transduction database. Nucleic Acids Res. 35, D386–D390. 10.1093/nar/gkl932 17135192 PMC1747179

[B207] Vázquez A. Dobrin R. Sergi D. Eckmann J.-P. Oltvai Z. N. Barabási A.-L. (2004). The topological relationship between the large-scale attributes and local interaction patterns of complex networks. *Proc. Natl. Acad. Sci*. U.S.A. 101, 17940–17945. 10.1073/pnas.0406024101 PMC539752 15598746

[B208] Wagner S. Wagner D. (2007). Comparing Clusterings - An Overview. Karlsruhe. Available online at: https://publikationen.bibliothek.kit.edu/1000011477 (accessed July 24, 2019).

[B209] Watts D. J. Strogatz S. H. (1998). Collective dynamics of “small-world” networks. Nature 393, 440–442. 10.1038/30918 9623998

[B210] Wernicke S. Rasche F. (2006). FANMOD: a tool for fast network motif detection. Bioinform. Oxf. Engl. 22, 1152–1153. 10.1093/bioinformatics/btl038 16455747

[B211] Xenarios I. Rice D. W. Salwinski L. Baron M. K. Marcotte E. M. Eisenberg D. (2000). DIP: the database of interacting proteins. Nucleic Acids Res. 28, 289–291. 10.1093/nar/28.1.289 10592249 PMC102387

[B212] Xu R. Wunsch D II.. (2005). Survey of clustering algorithms. IEEE Trans. Neural Netw. 16, 645–678. 10.1109/TNN.2005.845141 15940994

[B213] Yang Z. (1996). Phylogenetic analysis using parsimony and likelihood methods. J. Mol. Evol. 42, 294–307. 10.1007/BF02198856 8919881

[B214] Yevshin I. Sharipov R. Kolmykov S. Kondrakhin Y. Kolpakov F. (2019). GTRD: a database on gene transcription regulation-2019 update. Nucleic Acids Res. 47, D100–D105. 10.1093/nar/gky1128 30445619 PMC6323985

[B215] Yifan H. (2005). Efficient, high-quality force-directed graph drawing. Math. J. 10, 37–71. Available online at: http://asus.myds.me:6543/paper/ktall/37%20-%201984%20-%20Efficient,%20High-Quality%20Force-Directed%20Graph%20Drawing.pdf

[B216] Yook S.-H. Oltvai Z. N. Barabási A.-L. (2004). Functional and topological characterization of protein interaction networks. Proteomics 4, 928–942. 10.1002/pmic.200300636 15048975

[B217] Yue X. Wang Z. Huang J. Parthasarathy S. Moosavinasab S. Huang Y.. (2019). Graph embedding on biomedical networks: methods, applications and evaluations. Cowen L Ed. Bioinform. btz718. 10.1093/bioinformatics/btz718 31584634 PMC7703771

[B218] Zampelli S. Deville Y. Solnon C. (2010). Solving subgraph isomorphism problems with constraint programming. Constraints 15, 327–353. 10.1007/s10601-009-9074-3

[B219] Zhang H. Liang Y. Han S. Peng C. Li Y. (2019). Long noncoding RNA and protein interactions: from experimental results to computational models based on network methods. Int. J. Mol. Sci. 20:1284. 10.3390/ijms20061284 30875752 PMC6471543

[B220] Zhou C. (2016). A Survey of Edge Bundling Methods for Graph Visualization.

[B221] Zhou H. Panpan X.u Yuan X. Qu H. (2013). Edge bundling in information visualization. Tsinghua. Sci. Technol. 18, 145–156. 10.1109/TST.2013.6509098

