# Corrigendum: A guide to conquer the biological network era using graph theory

**DOI:** 10.3389/fbioe.2023.1182500

**Published:** 2023-03-31

**Authors:** Mikaela Koutrouli, Evangelos Karatzas, David Paez-Espino, Georgios A. Pavlopoulos

**Affiliations:** ^1^ Institute for Fundamental Biomedical Research, BSRC “Alexander Fleming”, Vari, Greece; ^2^ Department of Informatics and Telecommunications, University of Athens, Athens, Greece; ^3^ Lawrence Berkeley National Laboratory, Department of Energy, Joint Genome Institute, Walnut Creek, CA, United States

**Keywords:** biological networks, topology, graph theory, visualization, clustering

In the published article, there was an error in Figure 14 as published. The numbers in row V4 of the matrix in Figure 14A were inserted incorrectly. The corrected Figure 14 and its caption appear below.

**FIGURE 14:**
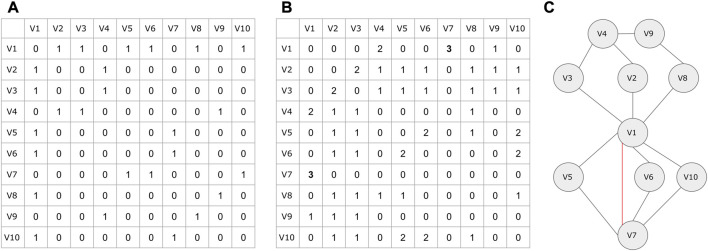
A triangle closing link prediction example. **(A)** The adjacency matrix of the undirected, unweighted example network. **(B)** The algebraic representation of the *A* ^2^ matrix. Each (*i,j*) value represents the number of common neighbors of the nodes *i* and *j*. **(C)** The example network plot. The red edge represents the new predicted link in time point *t* + *1*.

The authors apologize for this error and state that this does not change the scientific conclusions of the article in any way. The original article has been updated.

